# Association between Birth Characteristics and Cardiovascular Autonomic Function at Mid-Life

**DOI:** 10.1371/journal.pone.0161604

**Published:** 2016-08-23

**Authors:** Nelli Perkiömäki, Juha Auvinen, Mikko P. Tulppo, Arto J. Hautala, Juha Perkiömäki, Ville Karhunen, Sirkka Keinänen-Kiukaanniemi, Katri Puukka, Aimo Ruokonen, Marjo-Riitta Järvelin, Heikki V. Huikuri, Antti M. Kiviniemi

**Affiliations:** 1 Research Unit of Internal Medicine, Medical Research Center Oulu, Oulu University Hospital and University of Oulu, Oulu, Finland; 2 Center for Life Course Health Research, University of Oulu, Oulu, Finland; 3 Unit of Primary Health Care, Oulu University Hospital, Oulu, Finland; 4 Physiological Signal Analysis Team, Center for Machine Vision and Signal Analysis, University of Oulu, Oulu, Finland; 5 NordLab Oulu, Medical Research Center Oulu, Oulu University Hospital and Department of Clinical Chemistry, University of Oulu, Finland; 6 Department of Epidemiology and Biostatistics, MRC–PHE Centre for Environment & Health, School of Public Health, Imperial College London, London, United Kingdom; 7 Biocenter Oulu, University of Oulu, Oulu, Finland; University of Adelaide, AUSTRALIA

## Abstract

**Background:**

Low birth weight is associated with an increased risk of cardiovascular diseases in adulthood. As abnormal cardiac autonomic function is a common feature in cardiovascular diseases, we tested the hypothesis that low birth weight may also be associated with poorer cardiac autonomic function in middle-aged subjects.

**Methods:**

At the age of 46, the subjects of the Northern Finland Birth Cohort 1966 were invited to examinations including questionnaires about health status and life style and measurement of vagally-mediated heart rate variability (rMSSD) from R-R intervals (RRi) and spontaneous baroreflex sensitivity (BRS) in both seated and standing positions. Maternal parameters had been collected in 1965–1966 since the 16^th^ gestational week and birth variables immediately after delivery. For rMSSD, 1,799 men and 2,279 women without cardiorespiratory diseases and diabetes were included and 902 men and 1,020 women for BRS. The analyses were adjusted for maternal (age, anthropometry, socioeconomics, parity, gestational smoking) and adult variables (life style, anthropometry, blood pressure, glycemic and lipid status) potentially confounding the relationship between birth weight and autonomic function.

**Results:**

In men, birth weight correlated negatively with seated (r = -0.058, p = 0.014) and standing rMSSD (r = -0.090, p<0.001), as well as with standing BRS (r = -0.092, p = 0.006). These observations were verified using relevant birth weight categories (<2,500 g; 2,500–3,999 g; ≥4,000 g). In women, birth weight was positively correlated with seated BRS (r = 0.081, p = 0.010), but none of the other measures of cardiovascular autonomic function. These correlations remained significant after adjustment for potential confounders (p<0.05 for all).

**Conclusions:**

In men, higher birth weight was independently associated with poorer cardiac autonomic function at mid-life. Same association was not observed in women. Our findings suggest that higher, not lower, birth weight in males may contribute to less favourable cardiovascular autonomic regulation and potentially to an elevated cardiovascular risk in later life.

## Introduction

Impaired cardiovascular autonomic function increases susceptibility to cardiac events and death in various populations [1–3]. Abnormal autonomic function typically is manifested as depressed antiarrhythmic vagal activity and increased proarrhythmic sympathetic activity resulting in an increased risk of suffering fatal ventricular tachyarrhythmias and fibrillation [4]. Many factors are known to influence autonomic function, including common cardiometabolic risk factors and life style [5,6]. However, these factors explain surprisingly little of the inter-individual variance in cardiovascular autonomic function, suggesting not only the contribution of a large genetic component but also the presence of unknown determinants [7].

Low birth weight (BW) and impaired early growth have been related to an increased risk of developing various cardiovascular diseases and events [8–12]. While abnormal cardiac autonomic function is a common feature in major cardiovascular and metabolic diseases [13–15], little is known of the contribution of fetal and early life growth features to the development of cardiac autonomic dysregulation. With respect to heart rate variability (HRV), the relationship between early growth and cardiovascular autonomic function has proved controversial with some studies finding evidence for reduced cardiac vagal activity and a shift of autonomic balance towards a sympathetic predominance in children whose BW was <2.5 kg [16], whereas contrasting results have been reported when this relationship has been examined in adults [17]. Previous retrospective data also suggest that in young adults a low BW may be associated with poorer baroreflex sensitivity (BRS) [18].

The purpose of the present study was to determine the association of early growth and size at birth with cardiovascular autonomic function in men and women when they had reached middle-age (i.e. the age of 46). To address this question, we examined the Northern Finland Birth Cohort 1966 (NFBC 1966) which is a large prospective study with extensive data on these same individuals from the fetal period to mid-age. We hypothesized that a low BW may be associated with poorer cardiovascular autonomic function in adulthood.

## Materials and Methods

### Subjects

The subjects whose expected date of birth fell between January 1^st^ and December 31^st^ 1966 in the Northern Finland were included in the prospective NFBC 1966 study (96.3% of all births in 1966 in this area, n = 12,058 live births). Starting from their mothers’ recruitment during their first visit to maternity health centers on average on the 16^th^ gestational week, data have been collected on the subjects’ health, life style and socioeconomic status. The study has been conducted according to the Declaration of Helsinki and approved by the Ethical Committee of the Northern Ostrobothnia Hospital District in Oulu, Finland. The subjects have provided their written informed consent for the study.

### Birth and maternal variables

The early growth and maternal data have been described in detail elsewhere [11]. Briefly, maternal data were collected by questionnaire during the 24^th^ to 28^th^ gestational weeks. Paternal (maternal if mother was single) socioeconomic status, mother’s age, weight, height, body mass index, parity and maternal smoking status were established. Paternal socioeconomic status (high, middle, low, farmer), parity (1, 2–3 and ≥4) and maternal smoking status (smoking ≥1 cigarette/day after 2^nd^ month of pregnancy) were categorized using cutoffs adapted from Järvelin et al. 2004 [11]. For example, paternal socioeconomic categories were reduced to the most relevant four by merging the two highest categories, and the farmer categories. Birth weight (±5 g) and length (±1 cm) were measured immediately after delivery. Gestational age was determined from the mother’s last menstrual period. Using these variables, BW relative to gestational age and ponderal index (BW/length^3^) were calculated. Data about maternal weight gain were available for 903 mothers and were assessed as continuous variables and tertiles (<8, 8–12 and ≥13 kg). Birth weight (<2500, 2500–3999 and ≥4000 g, min-max: 1230–6080 g), birth length (<50, 50–51 and ≥51 cm, min-max: 35–59 cm), gestational age (<37, 37–41 and ≥42 weeks, min-max: 26–46 weeks) and percentiles of BW relative to gestational age (≤10, 11–90 and >90%) were also categorized using predefined cutoffs adapted from Järvelin et al. 2004 [11].

### Protocol at age of 46

A postal questionnaire on the participant’s health status and life style was conducted in 2012–2014. The questionnaires were mailed to all subjects whose addresses were known (n = 10,331). The response rate was 66% (n = 6,825). Subjects, who were living at known addresses in Finland, were invited to clinical examinations that were coordinated by the NFBC project center at the Center for Life Course Health Research (University of Oulu) with three laboratory units (Oulu, Southern and Northern Finland). A total of 5,861 (57%) subjects participated in clinical examinations between April 2012 and March 2014. The subjects entered the laboratory between 7:00 and 11:00 a.m. after an overnight fasting period (12 hours). Subjects were instructed to avoid smoking and drinking coffee during the examination day. Venous blood samples were drawn from an antecubital vein for the analysis of glycemic and lipid status. Serum glucose was analyzed using an enzymatic hexokinase/glucose-6-phosphate dehydrogenase method. Total cholesterol, high-density lipoprotein and low-density lipoprotein cholesterol, and triglycerides were determined using an enzymatic assay method. The concentration of glycated and total hemoglobin were measured using immunochemical assay methods. The ratio is reported as percent hemoglobin A1c (NGSP). The samples were analyzed in NordLab Oulu, a testing laboratory (T113) accredited by Finnish Accreditation Service (FINAS) (EN ISO 15189) (All methods: Advia 1800; Siemens Healthcare Diagnostics Inc., Tarrytown, NY, USA). Systolic (SBP) and diastolic blood pressure (DBP) were measured three times in 1 minute periods (the two lowest systolic values and the corresponding diastolic values averaged) with an automated sphygmomanometer (Omron M10, Omron Healthcare, Kyoto, Japan) in a sitting position from the right arm after 15 minutes of rest. After various measurements, including the measurements of weight, height and circumferences of waist and hip, the participants had a light meal 60–90 min before the assessments of cardiovascular autonomic function. On a separate day after an overnight fasting period (12 hours), a 2-hour oral glucose tolerance test was conducted for these participants without medication for diabetes according to the recommendations [19].

#### Measurement of cardiovascular autonomic function

The participant sat on a chair for instrumentation and the review of the protocol. A heart rate (HR) monitor (RS800CX, Polar Electro Oy, Kempele, Finland) was used to record R-R intervals (RRi) with an accuracy of 1 ms. In about half of the participants (Oulu laboratory unit only) spontaneous BRS was also assessed. Standard lead-II ECG (Cardiolife, Nihon Kohden, Tokyo, Japan), breathing frequency (MLT415/D, Nasal Temperature Probe, ADInstruments, Bella Vista, New South Wales, Australia), and blood pressure (BP) by finger plethysmography (Nexfin, BMEYE Medical Systems, Amsterdam, the Netherlands) were recorded during the protocol with a sampling frequency of 1,000 Hz (PowerLab 8/35, ADInstruments). The finger cuff was adjusted so that the values of SBP and DBP assessed by finger plethysmography (left arm) did not differ by more than 10 mmHg from those measured by automated sphygmomanometer at the same time (right arm, Omron M10). Physiological calibration of finger plethysmography was then turned off. An arm sling was used to support the left arm during the measurements. These procedures which were conducted in the seated position lasted for a total of 5–10 min which was followed by stabilization of HR for at least 1 min before the recording started. After 3-min recording in a seated position, the participant stood up and remained still in a standing position for 3 min while breathing spontaneously. The first 150 s of recording in seated position and the last 150 s in standing position were analyzed.

#### Analysis of heart rate variability

The RRi data were edited based on visual inspection (Hearts 1.2, University of Oulu, Oulu, Finland). Artefacts and ectopic beats were removed and replaced by local average. However, sequences with ≥10 consecutive beats of noise or ectopic beats were deleted. The RRi series with ≥80% accepted data were included in analyses. A total of 5,679 subjects attended RRi recordings of whom 5,473 (96%) had eligible HRV data for both phases of the protocol (seated and standing). Mean HR, root mean square of successive differences in RRi (rMSSD, ms), spectral power densities (fast Fourier transform, length 512 beats) at low (LF, 0.04–0.15 Hz, ms^2^) and high frequency (HF, 0.15–0.40 Hz, ms^2^) components of HRV, and their ratio (LF/HF) were analyzed [20]. Additionally, rMSSD was divided by 3^rd^ power of RRi (s) to remove RRi-dependency of rMSSD [21].

#### Analysis of baroreflex sensitivity

Continuous ECG, BP and respiration signals were imported into custom-made standalone Matlab-based software (Biosignal Processing Team, University of Oulu, Oulu, Finland). RRi and SBP values were extracted from the continuous ECG and BP recordings as discrete event series. Artefacts and ectopic beats were replaced using linear interpolation (<5% for accepted recording) and, thereafter, resampled at 2 Hz. Very-low-frequency components (< 0.04 Hz) were removed using the Savitzky-Golay method. A fast Fourier transform (Welch method, segments of 128 samples with 50% overlap, length 1024 samples) was performed to analyze the LF power of RRi and SBP oscillations (ms^2^, mmHg^2^) for subsequent analysis of BRS by the alpha method if sufficient coherence (≥0.5) between LF oscillations in RRi and SBP was verified [22,23]. Out of 2,726 recordings, BRS was successfully calculated for 2,641 subjects in the seated position and 2,617 while they were standing. The main reasons why BRS could not be analyzed were numerous ectopic beats and technical artefacts.

#### Life style factors at age 46

Based on their answers in the postal questionnaire, subjects were defined as current smokers if they smoked regularly on ≥2 days/week. The amount of alcohol consumed per day was estimated from the questions measuring the frequency and the usual amount of beer, wine and spirits consumed on one occasion. The subjects were then categorized into two groups based on the highest sex-specific deciles rounded to the closest 10 g/d (cut-off for men: 40 g/d, women: 20 g/d). Total sitting time during waking hours was established by asking the subjects how many hours on average they sat on weekdays and the responses were dichotomized (cut-off 11 h/d). To evaluate the sufficiency of sleep, the subjects were asked how tired they felt in the morning during the first half an hour (very/somewhat tired = tired, somewhat/well rested = rested). In the estimation of physical activity, the subjects were asked how often they participated in brisk physical activity/exercise during their leisure-time. The term 'brisk' was defined as physical activity causing at least some sweating and getting out of breath, corresponding to moderate-to-vigorous intensity. Six response alternatives were 1) daily, 2) 4–6 times a week, 3) 2–3 times a week, 4) once a week, 5) 2–3 times a month, and 6) once a month or less often. Three relevant classes were formed by combining two consecutive categories (1+2, 3+4 and 5+6).

### Inclusions/Exclusions

In those individuals for whom there was HRV data (n = 5,473), 255 subjects with diabetes based on previous or new diagnosis according to the criteria issued by the World Health Organization (fasting plasma glucose ≥7.0 mmol/l or 2-hour glucose ≥11.1 mmol/l in oral glucose test or glycated hemoglobin ≥6.5%) were excluded [19]. In addition, subjects with cardiac (n = 88) or respiratory disease (n = 246) or who were on antihypertensive medication (n = 570) were excluded. Therefore, the maximum population for birth variable-specific analysis included 1,799 men and 2,279 women for HRV, 909 men and 1,024 women for seated BRS and 902 men and 1,020 women for standing BRS.

### Statistical analysis

The distributions of the dependent variables were assessed by analyzing the skewness of the data and by visual inspection of histograms. In the case of skewed distributions (|skewness| >1 [24], all dependent variables except for mean HR), the variable was transformed into natural logarithm (ln), which eliminated skewness in the dependent variables. These transformed variables were visually verified for Gaussian. (S1 Table, S1 File). Pearson’s correlation analysis was used to assess the relationships between continuous birth variables and markers of cardiac autonomic function. The associations between categorized birth variables and cardiac autonomic function were tested by one-way ANOVA followed by Bonferroni post hoc test when appropriate.

All significant associations between an early growth variable and cardiac autonomic function in univariate analysis (Pearson’s correlation or one-way ANOVA) were further adjusted in multiple regression models (continuous early growth variables) or ANCOVA (categorized early growth variables), first, for maternal variables (socioeconomics, age, height, weight, body mass index, smoking after 2^nd^ month of pregrancy and parity), and, subsequently, also for continuous adult anthropometric (body mass index, weight, height and waist-hip ratio) and cardiometabolic variables (SBP, DBP, glycated hemoglobin, glucose, total cholesterol, high and low density cholesterol and triglycerides) as well as categorized adult life style variables (current smoking, sitting time, alcohol consumption, sufficiency of sleep and physical activity). Maternal weight, adult weight and low density cholesterol were excluded from the independent variables list to eliminate collinearity (VIF<5 for all independent variables in the final models, S2 Table). Second degree polynomial terms were added for maternal age, height and BMI to account for their nonlinear relationships to the dependent variables. Visual inspection verified all model residuals as Gaussian. Among the included subjects, there were missing data in independent variables and covariates. The variation in the number of subjects in specific analyses are noted in the results. The data were analyzed using SPSS software (IBM SPSS Statistics 21, IBM Corp., New York, USA). A p-value <0.05 was considered as statistically significant.

## Results

### Birth characteristics and autonomic function at mid-life in men

**Fig 1:**
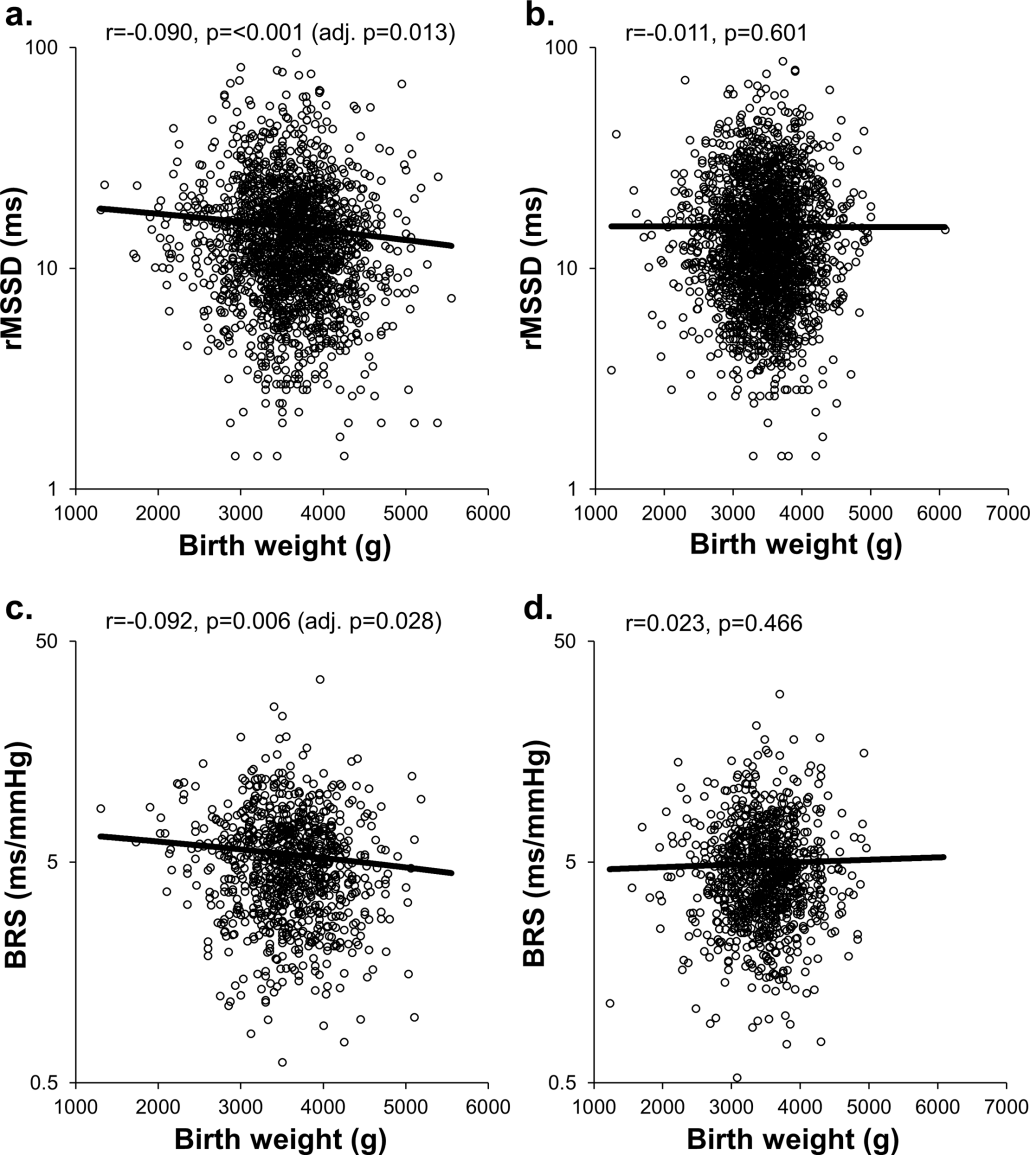
**Scattergrams presenting the distribution of standing rMSSD and BRS according to birth weight in men (a, c) and women (b, d).** Pearson correlation (r) and p-values as well as p-values adjusted for maternal and adult variables are presented. Y-axis is log-scaled.

**Fig 2:**
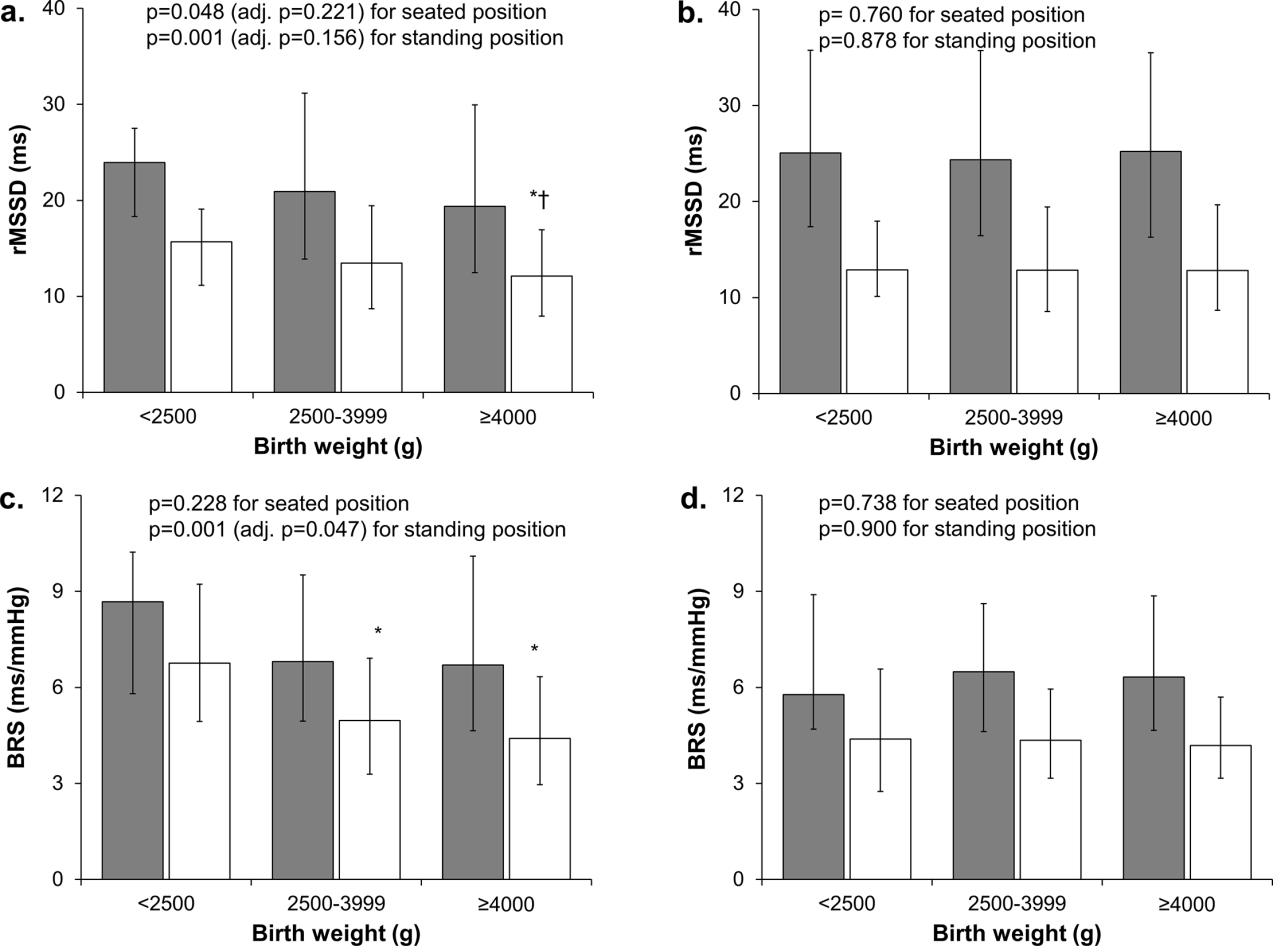
**rMSSD and BRS in seated (grey) and standing (white) positions in different birth weight groups in men (a, c) and women (b, d).** Values are medians and error bars represent 1^st^-3^rd^ quartiles. Univariate (ANOVA) p-values and p-values adjusted for maternal and adult variables (ANCOVA) are presented. * p<0.05 vs. birth weight <2500 g group, † p<0.05 vs. birth weight 2500–3999 g group.

The characteristics of the cohort are presented in Table 1. Table 2 presents all significant univariate associations between birth variables and autonomic markers in men. Birth weight correlated negatively with rMSSD and rMSSD/RRi^3^ in seated and standing positions as well as BRS in standing position (Table 2, Fig 1A and 1C), and positively with standing HR. Significant differences were also observed between BW categories in seated and standing rMSSD and standing rMSSD/RRi^3^ and BRS (Table 2, Fig 2A and 2C). The highest BW category had significantly lower standing rMSSD compared to the middle (p = 0.002) and lowest (p = 0.015) BW categories. Also, the highest (p = 0.001) and middle (p = 0.008) BW categories had significantly lower measures of BRS than the lowest BW category. The BW relative to gestational age as a categorized variable associated with standing BRS, with the highest group having significantly lower BRS compared to the lowest group (p = 0.021). Gestational age correlated negatively with standing rMSSD/RRi^3^. Also, birth length correlated negatively with standing rMSSD and BRS, and positively with standing HR. Ponderal index correlated negatively with rMSSD and rMSSD/RRi^3^ in seated and standing positions and positively with standing LF/HF. Finally, twins had higher measures of sitting and standing rMSSD/RRi^3^ than singletons.

**Table 1:** Characteristics of the study population.

	Men	Women
**Birth variables**		
Birth weight, g	3591 (542)	3455 (494)
<2500 g, n	52 (3%)	71 (3%)
2500–3999 g, n	1359 (76%)	1924 (84%)
≥4000 g, n	388 (22%)	284 (13%)
Birth weight-Gestational age, n		
≤10%	189 (11%)	268 (12%)
11–90%	1417 (79%)	1774 (78%)
>90%	193 (11%)	237 (10%)
Birth length,cm	50.9 (2.1)	50.0 (2.0)
Gestational age, weeks	40.1 (1.9)	40.1 (1.9)
Ponderal index, kg/m^3^	27.2 (2.5)	27.6 (2.5)
Multiple birth, n	43 (2%)	41 (2%)
Gestational weight gain, kg*	10.4 (4.6)	10.1 (4.5)
Life style at 46 years**		
Current smoker, n	310 (19%)	313 (14%)
Alcohol consumption ≥40/20 g/d (♂/♀), n	144 (9%)	161 (7%)
Insufficient sleep, n	499 (30%)	687 (32%)
Sitting time ≥ 11h/d, n	260 (16%)	194 (9%)
Physical activity, n		
Active	271 (16%)	372 (17%)
Semi-active	919 (56%)	1298 (60%)
Inactive	452 (28%)	486 (23%)
**Laboratory measurements at 46 years**		
SBP, mmHg	128 (14)	117 (15)
DBP, mmHg	85 (10)	81 (10)
BMI, kg/m^2^	26.7 (3.8)	25.7 (4.6)
Waist-hip-ratio	0.97 (0.06)	0.86 (0.06)
HbA1c, %	5.47 (0.34)	5.39 (0.35)
Plasma glucose, mmol/L	5.59 (0.47)	5.26 (0.46)
Total cholesterol, mmol/L	5.58 (0.97)	5.16 (0.85)
LDL cholesterol, mmol/L	3.77 (0.93)	3.20 (0.85)
HDL cholesterol, mmol/L	1.42 (0.34)	1.70 (0.40)
Triglycerides, mmol/L	1.41 (0.89)	1.01 (0.49)
**Cardiac autonomic function at 46 years**		
Heart rate_Sit_, bpm	70 (63–78)	71 (65–78)
Heart rate_Stand_, bpm	80 (72–90)	82 (75–91)
rMSSD_Sit_, ms	20.6 (13.7–30.6)	24.4 (16.5–35.7)
rMSSD_Stand_, ms	13.2 (8.6–18.9)	12.8 (8.6–19.3)
rMSSD/RRi^3^_Sit_	31.8 (24.7–42.2)	39.8 (30.2–52.1)
rMSSD/RRi^3^_Stand_	30.2 (23.4–40.1)	32.4 (24.7–43.2)
LF/HF_Sit_	2.31 (1.26–4.03)	1.23 (0.70–2.27)
LF/HF_Stand_	4.53 (2.49–7.63)	2.66 (1.52–4.85)
BRS_Sit_, ms/mmHg***	6.82 (4.94–9.60)	6.46 (4.63–8.65)
BRS_Stand_, ms/mmHg***	4.91 (3.29–6.87)	4.33 (3.16–5.96)

Values are mean (SD), median (1^st^-3^rd^ quartile) or n (%). *SBP* systolic, *DBP* diastolic blood pressure, *BMI* body mass index, *HbA1c* glycated hemoglobin, *LDL* low-density lipoprotein, *HDL* high-density lipoprotein, *rMSSD* root mean square of the successive differences in R-R intervals, *RRi* R-R interval, *BRS* baroreflex sensitivity, *LF* low frequency power of RRi oscillations (0.04–0.15 Hz), *HF* high frequency power of RRi oscillations (0.15–0.4 Hz). n = 1,744–1,799 for men and n = 2,196–2,279 for women unless noted otherwise.

*n = 395/508

**n = 1,642–1,672/2,156–2,182, and

***n = 902/1,020 for men/women.

**Table 2:** Birth variables as determinants of autonomic function adjusted for maternal and adult variables in men. All significant univariate associations are presented followed by adjustments for maternal and adult variables.

	Univariate			Multivariate					
				Adj. for maternal variables**			Adj. for maternal and adult variables***		
	Birth variables	r	p	R^2^ for model	Beta/η^2^*	p	R^2^ for model	Beta/η^2^*	p
***Sitting***									
**RMSSD**	Birth weight	-0.058	0.014	0.023	-0.071	0.007	0.225	-0.060	**0.021**
	Birth weight (Cat.)	-	0.048	0.022	0.004*	0.055	0.224	0.002*	0.221
	Ponderal index	-0.050	0.035	0.021	-0.057	0.024	0.227	-0.072	**0.003**
**RMSSD/RRI**^**3**^	Birth weight	-0.053	0.023	0.014	-0.068	0.010	0.065	-0.043	0.135
	Ponderal index	-0.062	0.008	0.015	-0.071	0.005	0.071	-0.087	**0.001**
	Multiple birth	-	<0.001	0.016	0.006*	0.002	0.070	0.006*	**0.003**
***Standing***									
**HR**	Birth weight	0.051	0.032	0.015	0.045	0.084	0.184	0.040	0.136
	Birth length	0.052	0.028	0.014	0.043	0.096	0.185	0.032	0.230
**RMSSD**	Birth weight	-0.090	<0.001	0.025	-0.083	0.001	0.158	-0.068	**0.013**
	Birth weight (Cat.)	-	0.001	0.024	0.006*	0.008	0.157	0.003*	0.156
	Birth length	-0.064	0.007	0.021	-0.051	0.050	0.157	-0.015	0.573
	Ponderal index	-0.062	0.009	0.022	-0.060	0.019	0.163	-0.078	**0.002**
**RMSSD/RRI**^**3**^	Birth weight	-0.079	0.001	0.017	-0.073	0.005	0.056	-0.061	**0.034**
	Birth weight (Cat.)	-	0.003	0.016	0.005*	0.015	0.055	0.003*	0.132
	Gestational age	-0.067	0.005	0.016	-0.069	0.006	0.059	-0.063	**0.018**
	Ponderal index	-0.070	0.003	0.015	-0.068	0.007	0.059	-0.091	**0.001**
	Multiple birth	-	0.018	0.013	0.002*	0.059	0.055	0.003*	0.051
**LF/HF**	Ponderal index	0.052	0.027	0.012	0.042	0.096	0.042	0.057	**0.038**
**BRS**	Birth weight	-0.092	0.006	0.038	-0.078	0.035	0.251	-0.080	**0.028**
	Birth weight (Cat.)	-	0.001	0.043	0.010*	0.016	0.252	0.009*	**0.047**
	Birth length	-0.084	0.012	0.022	-0.074	0.044	0.248	-0.048	0.196
	Birth weight-Gest.age (Cat.)	-	0.020	0.037	0.004*	0.175	0.247	0.002*	0.577

The values are Pearson’s correlation coefficients (r), statistical significances (linear regression for continuous birth variables, ANCOVA for categorized birth variables) (p), explained variance of the model (R^2^), and standardized beta (Beta) or partial ETA squared (η^2^) (* = η^2^, for categorized birth variables) for the main independent variable. *HR* heart rate, *rMSSD* root mean square of the successive differences in R-R intervals, *RRi* R-R interval, *LF* low frequency power of RRi oscillations (0.04–0.15 Hz), *HF* high frequency power of RRi oscillations (0.15–0.4 Hz), *BRS* baroreflex sensitivity at low frequency by alpha method, *Cat*. categorized.

**Maternal variables: father’s socioeconomic status, maternal age, height, body mass index and smoking after 2^nd^ month of pregnancy and parity. Maternal age, height and body mass index 2^nd^ degree continuous variables, other maternal variables categorized. n = 1,592–1,629/1,989–2,054 for men/women except for BRS (n = 813-820/924-931 for men/women).

***Continuous adult variables: body mass index, height, systolic and diastolic blood pressure, waist-hip ratio, glucose, glycated hemoglobin, total cholesterol, high density cholesterol, triglycerides. Categorized adult variables: current smoking, sitting time, alcohol consumption, sufficiency of sleep, physical activity. n = 1,388–1,422/1,807–1,865 for men/women except for BRS (n = 723-730/840-845 for men/women).

Except for standing HR, all associations between continuous BW and the measures of cardiac autonomic function remained significant after adjustment for maternal variables (Table 2). After further adjustment for adult variables, seated and standing rMSSD and standing BRS and rMSSD/RRi^3^ were significantly related to continuous BW. Categorized BW remained significantly associated with only standing BRS after adjustments. Also, ponderal index was related to rMSSD and rMSSD/RRi^3^ in seated and standing positions as well as standing LF/HF independently of maternal and adult variables. Finally, multiple birth remained a significant determinant of rMSSD/RRi^3^ in seated position and gestational age of rMSSD/RRi^3^ in standing position.

### Birth characteristics and autonomic function at mid-life in women

In women, BW was associated with seated BRS (Pearson r = 0.081, p = 0.010). Categorized BW had no significant association with cardiac autonomic function. Categorized birth length (<50 cm, 50–51 cm, >51 cm) showed nonlinear association with standing rMSSD (one-way ANOVA p = 0.038), with the 2^nd^ tertile having the lowest mean (p = 0.020 vs. 1^st^ tertile). Furthermore, birth length was positively related to standing LF/HF–ratio (Pearson’s r = 0.042, p = 0.043 for continuous).

In multivariate analysis, BW remained a significant determinant of seated BRS after adjustment for maternal (Beta = 0.092, p = 0.008, R^2^ for model = 0.029, n = 934) as well as maternal and adult variables (Beta = 0.071, p = 0.047, R^2^ for model = 0.176, n = 738). Birth length did not remain a significant factor underlying the standing LF/HF-ratio (Beta = 0.029, p = 0.208, R^2^ for model = 0.008, n = 2,036) or standing rMSSD (η^2^ = 0.003, p = 0.072, R^2^ for model = 0.008, n = 2,038) after adjustment for maternal variables.

### Maternal overweight and gestational weight gain

The effect of maternal overweight (BMI ≥25) on adult cardiovascular autonomic function of the offspring was also assessed. Univariate analysis (one way ANOVA) showed that the male offspring of overweight mothers had significantly lower measures of BRS in seated (p = 0.006) and standing (p = <0.001) positions and a similar tendency was observed with rMSSD (seated p = 0.060, standing p = 0.066). Maternal overweight was also associated with higher seated HR (p = 0.039) in men. In women, maternal overweight was associated with lower seated LF/HF (p = 0.015). As a continuous variable, maternal pregestational BMI was not related to adult cardiovascular autonomic function in men or in women. Maternal BMI and BW of offpring were significantly correlated in men (Pearson r = 0.208, p = <0.001) and in women (Pearson r = 0.204, p = <0.001).

In a subsample with gestational weight gain data, higher gestational weight gain tertile was related to lower standing rMSSD and higher seated and standing HR (p<0.05 for all) in men. As a continuous variable gestational weight gain was positively associated with seated LF/HF ratio (r = 0.100, p = 0.047) in men. After adjustments for maternal and adult variables, tendencies with LF/HF ratio (p = 0.050) and rMSSD (p = 0.086) remained. In women, a greater gestational weight gain was related to lower seated rMSSD and higher seated LF/HF ratio when assessed as a categorized (p = 0.015 for rMSSD, p = 0.015 for LF/HF) and as a continuous (r = -0.096, p = 0.030 for rMSSD, r = 0.095, p = 0.032 for LF/HF) variable. Continuous gestational weight gain remained a significant determinant of rMSSD (p = 0.045) after adjustments in women. A significant correlation between gestational weight gain and BW was observed in men (Pearson r = 0.241, p = <0.001) and in women (Pearson r = 0.206, p = <0.001).

## Discussion

The principal finding of the present study was that BW was independently negatively associated with cardiac vagal activity and BRS at mid-life in men. In women, however, a similar association between BW and later cardiovascular function was not observed, the association between BW and seated BRS being even opposite. The findings in men were contradictory to our primary hypothesis and suggest that, instead of impaired prenatal growth, greater prenatal growth contributes to lesser cardioprotective vagal activity in mid-life.

### Birth weight and cardiovascular risk

Several studies have detected an increased risk of developing various cardiovascular diseases and events in subjects with low BW [8,10–12]. Less has been reported about the long-term effects of high BW. Some studies have proposed that there may be a link between a high BW and obesity, high blood pressure and increased risk of atrial fibrillation in later life [25–27]. Boney et al. reported an increased risk of metabolic syndrome if the subject had a high BW in combination with maternal gestational diabetes mellitus (GDM) or obesity [28]. The U-shaped association between birth weight and the subsequent risk of type 2 diabetes [29] and the strengthening of the inverse association between BW and risk for adult cardiovascular disease when excluding those born large (> 4kg) [30] are evidence suggesting that the relationship between BW and cardiovascular risk may not be exclusively negative.

### Birth weight and autonomic function

Even though the associations of BW and cardiac autonomic function to cardiovascular morbidities have been studied extensively, surprisingly little is known about the potential link between BW and autonomic function in later life. Furthermore, most of these studies have discussed the effects of low BW, and thus the effect of high BW on autonomic function has not been examined to the same extent. The studies exploring the association between BW and HRV in infants or children have reported impaired cardiac vagal or overall autonomic activity in subjects with low BW [16,31,32]. In adolescence or adulthood, a low BW has been linked with increased sympathetic activity and/or depressed vagal activity, as assessed by increased HR and muscle sympathetic nerve activity as well as a shortened cardiac pre-ejection period in subjects with low BW [33–35]. In contrast, some studies have reported increased cardiac vagal activity and lower sympathetic nerve activity in adults with low BW [17,36], supporting our observations in vagally mediated HRV and BRS among men. However, in the present data, the differences were mainly observed between the low and high BW groups, not between low and normal BW groups.

Very few studies have examined the relationship between early growth and the properties of the baroreflex; in them, a positive association was observed between BW and BRS [18,37]. Therefore, our results in men are unexpected in this respect. In addition to autonomic tone, another important factor influencing the baroreflex-mediated autonomic outflow is the sensitivity of the baroreceptors, a property that is influenced by characteristics of the vascular wall, such as intima media thickness (IMT) in the carotid bulb [38]. Increased carotid IMT has been observed in young adults born large for gestational age [25], and this is a potential factor which may account for our findings. Although increased carotid IMT has been reported also in newborns with low BW, this association has not been observed in later life in several large studies [39]. Finally, it is noteworthy to remark, that although the negative association between BW and BRS in our study was clear, very few of our study subjects had BRS values <3 ms/mmHg (e.g. 54 men and 59 women while seated), which is the most commonly used definition for abnormal BRS in patient and population-based samples [2].

Our findings were sex-specific, with greater BW associating with lesser cardiac vagal activity and BRS at mid-life in men but not in women. Most studies examining the relationship between BW and autonomic function have not reported sex-specific results. There are some reports documenting a relationship between size at birth and later autonomic function in women but not in men [37]. Interestingly, we observed an independent significant positive association between seated BRS and BW in women, which underscores the sex-specific association between early growth and autonomic function in later life. However, this type of association was not uniformly observed between BW and other markers of cardiac autonomic function including standing BRS, presumably a more robust measure baroreflex physiology [40]. While we cannot explain the sex-specificity of our findings, it remains an important question to be answered by future studies.

### Potential mechanisms

Maternal hyperglycaemia in untreated pre-gestational diabetes and GDM, maternal pre-pregnancy obesity and excess weight gain during pregnancy appear to be independent risk factors for high BW in offspring [41–44] and may be potential mechanisms behind the findings of the present study. Furthermore, these factors are often associated with each other and may further increase the risk of high BW when more than one of them is present [41,43,44]. Maternal obesity has been previously reported to influence many cardiometabolic risk factors in adulthood [45], which is supported by our findings that the offspring of overweight mothers had lower measures of BRS and a similar tendency in rMSSD in univariate analysis in adulthood in men. In our data, also gestational weight gain tended to associate to measures of cardiac autonomic function, which is in line with previous findings suggesting high gestational weight gain to be associated with cardiometabolic risk factors in adulthood [45,46]. Data about gestational weight gain were only available for 22% of the study population and thus this must be taken into account when considering the results. In the 1960´s when the present study population was born, screening for GDM was not a part of clinical practice and, unfortunately not applied in the NFBC 1966. Therefore, we were not able to test our hypothesis on maternal GDM and autonomic function at mid-life. However, as the diagnosis of diabetes and GDM was not as strictly controlled as today, cases of undiagnosed and untreated DM are very likely present in mothers of our study population.

Ponderal index expresses body weight relative to length and thus is often used to describe the ‘thinness’ or ‘adiposity’ of newborns [47]. Our results showed that, in addition to birth weight, ponderal index was negatively associated with cardiac autonomic function at mid-life in men, suggesting that ‘thinner’ newborns had higher measures of cardiac autonomic function than newborns with a high ponderal index (macrosomic infants). A high ponderal index has also been shown to be related to a higher gestational weight gain in obese mothers [48], and to the risk of being overweight in adulthood in men [49]. However, one must acknowledge that ponderal index does not describe the composition of weight or differentiate between short and light compared to long and heavy newborns. It is also noteworthy that gestational age and BW relative to gestational age were related, but not consistently, to autonomic measures supporting the results obtained with BW in men. Therefore, the independent value of gestational age in respect to autonomic function in later life seems to be evidently lesser than BW.

### Methodological considerations

Our main results of BRS were observed in BRS measured in the standing position, corroborating evidence that the spontaneous BRS method reflects baroreflex physiology particularly if measured when the subject is standing [40]. Furthermore, the assessment of BRS seems to be more reproducible when measured during standing, the autonomic response potentially masking some interfering factors that could have a greater impact when BRS is measured in either the supine or seated position [50]. The measures of HRV are physiologically and mathematically dependent of average HR [21]. In the present study, we aimed to study whether BW exerted an impact on vagally mediated HRV (rMSSD) irrespective of mean HR by removing HR-dependency (both physiological and mathematical) by utilizing the specific power of RRi [21]. It was demonstrated that normalized HRV was largely related to many early growth factors but the model involving remarkably lesser explained variance (R^2^). However, our results suggest that BW makes a specific contribution to vagal modulation irrespective of prevailing mean HR.

### Limitations

The short recording period of RRi and BP may create a possible measurement bias, since the reproducibility of short-term recordings is weaker than that of longer recordings. For example, cardiovascular autonomic function is affected by the time from the previous meal, which although controlled, was relatively short in the present study. This was a tradeoff related to logistics and other competing priorities during these extensive series of measurements. Also, nicotine and caffeine withdrawal may have affected the measures of cardiac autonomic function. Spontaneous breathing may confound the spectral analysis of HRV and BRS [51]. In subjects with breathing signal (BRS data), only ~10% had breathing frequency at LF. Moreover, rMSSD is less vulnerable to slow breathing than the spectral indices. Gestational weight gain was only available for a limited part of the study population and thus a selection bias may be present when compared to the whole study group. Also, small groups based on commonly used cut-offs, for example, for low birth weight (<2500 g) and preterm birth (gestational age <37 weeks) limits the interpretation of some categorized variables but provide important reference to previous and potential future studies. It must be acknowledged that the NFBC1966 population measured at age of 46 did not fully represent the whole cohort regarding BW, which was 117 g greater in men and 127 g in women among those who participated in the follow-up measurements compared to those who did not.

## Conclusions

A higher birth weight was associated with lower cardiac vagal activity and reduced baroreflex sensitivity at mid-life in men, regardless of potential confounding effects of maternal variables and mid-life life style and cardiometabolic factors. In women, the effect of early growth parameters to cardiovascular autonomic function was not clear. These findings suggest that greater, not depressed, prenatal growth may contribute to poorer cardiovascular autonomic regulation and the related cardiac risk in later life in men.

## Supporting Information

S1 File Density plots of cardioautonomic variables before and after ln-transformation. (ZIP)

S1 Table Skewness and kurtosis values of cardioautonomic variables. (PDF)

S2 Table Multicollinearity diagnostics for statistical models. (DOCX)
