# Supplementary figures and images for: Association between Birth Characteristics and Cardiovascular Autonomic Function at Mid-Life
Source: PLoS One. 2016 Aug 23;11(8):e0161604. doi: 10.1371/journal.pone.0161604 (PMC4994955; doi:10.1371/journal.pone.0161604)

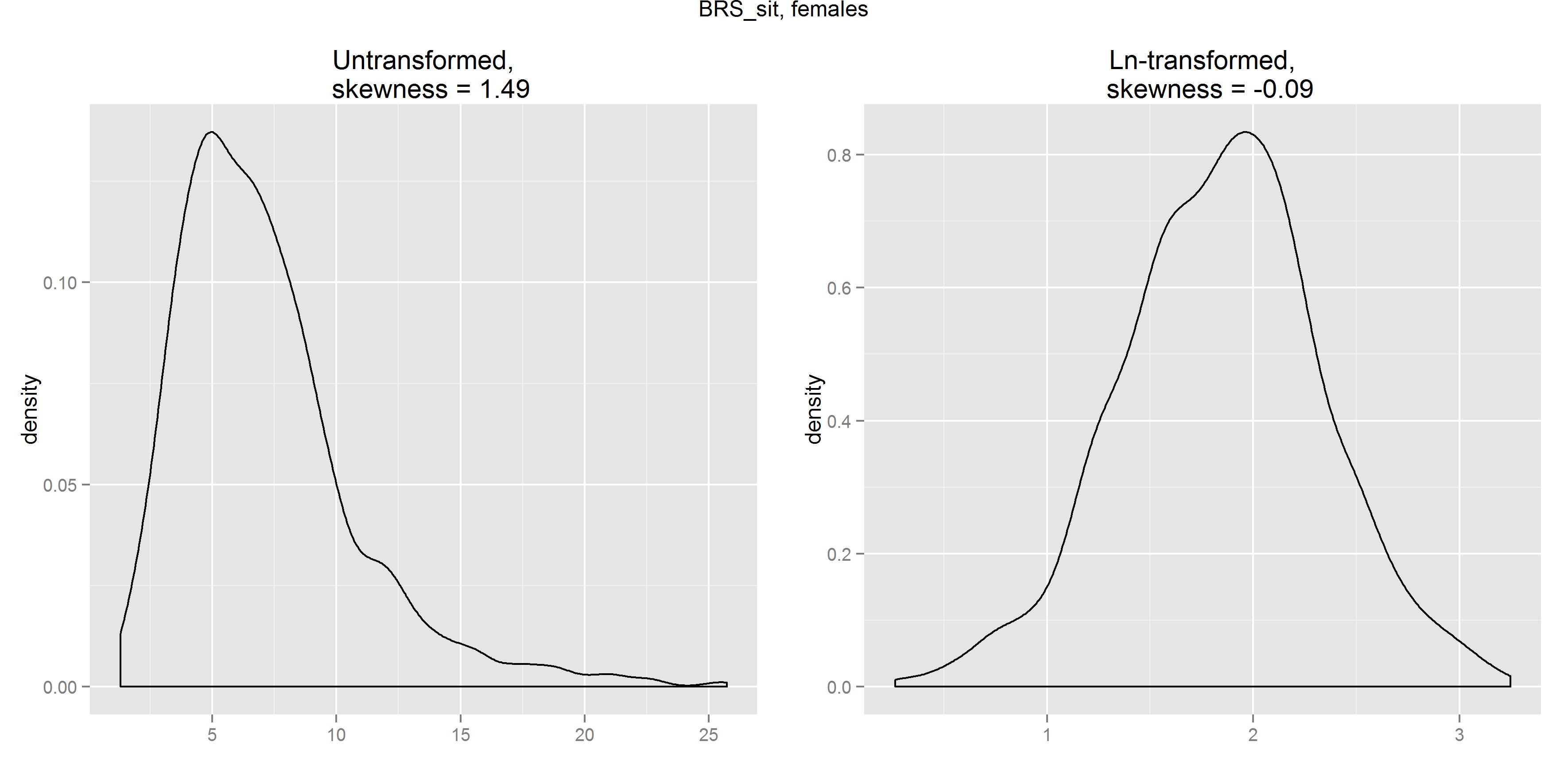

Supplement: S1 File — (ZIP) [file pone.0161604.s001.zip › BRS_sit_females_transformation_effect.jpg]

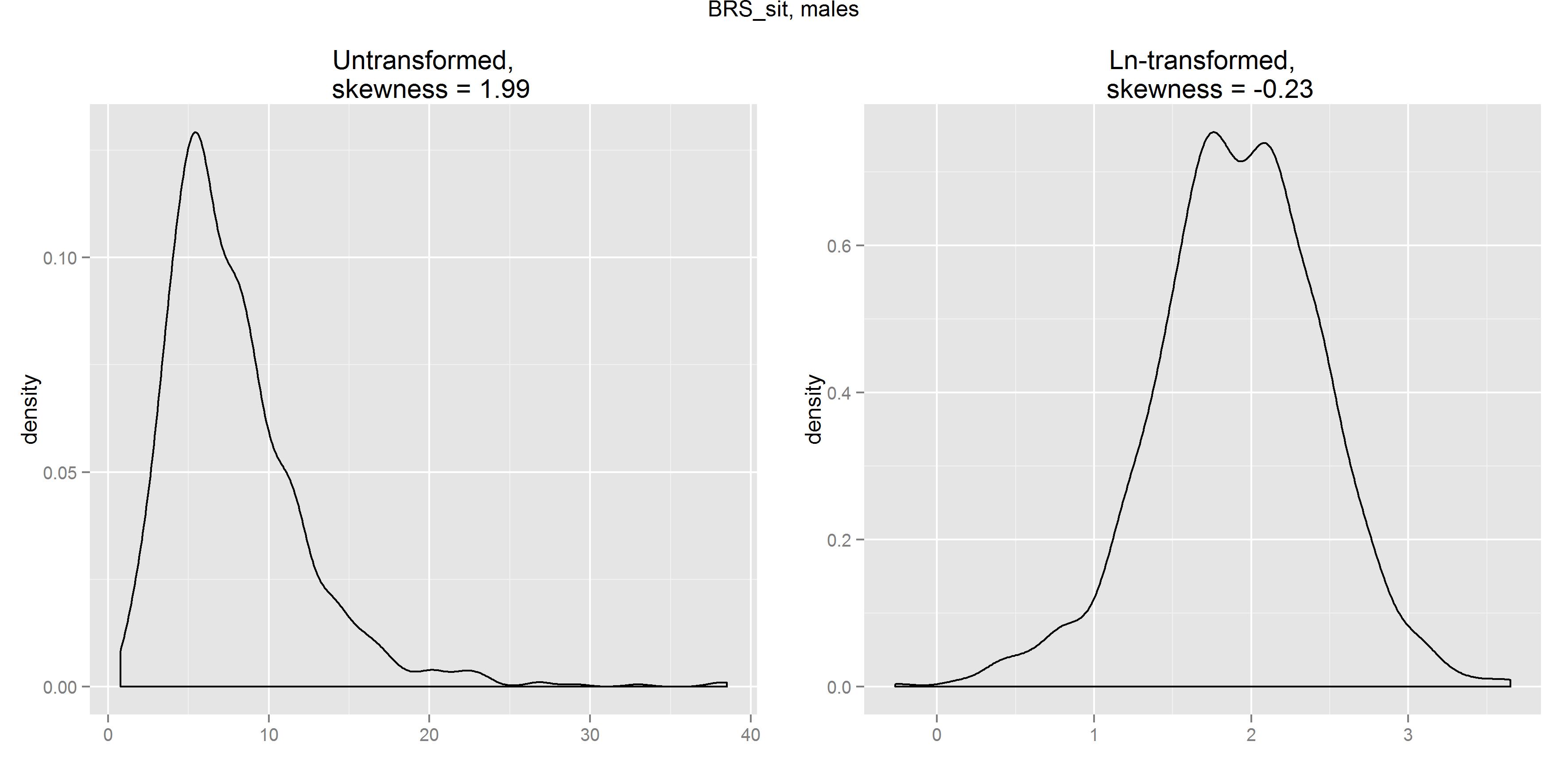

Supplement: S1 File — (ZIP) [file pone.0161604.s001.zip › BRS_sit_males_transformation_effect.jpg]

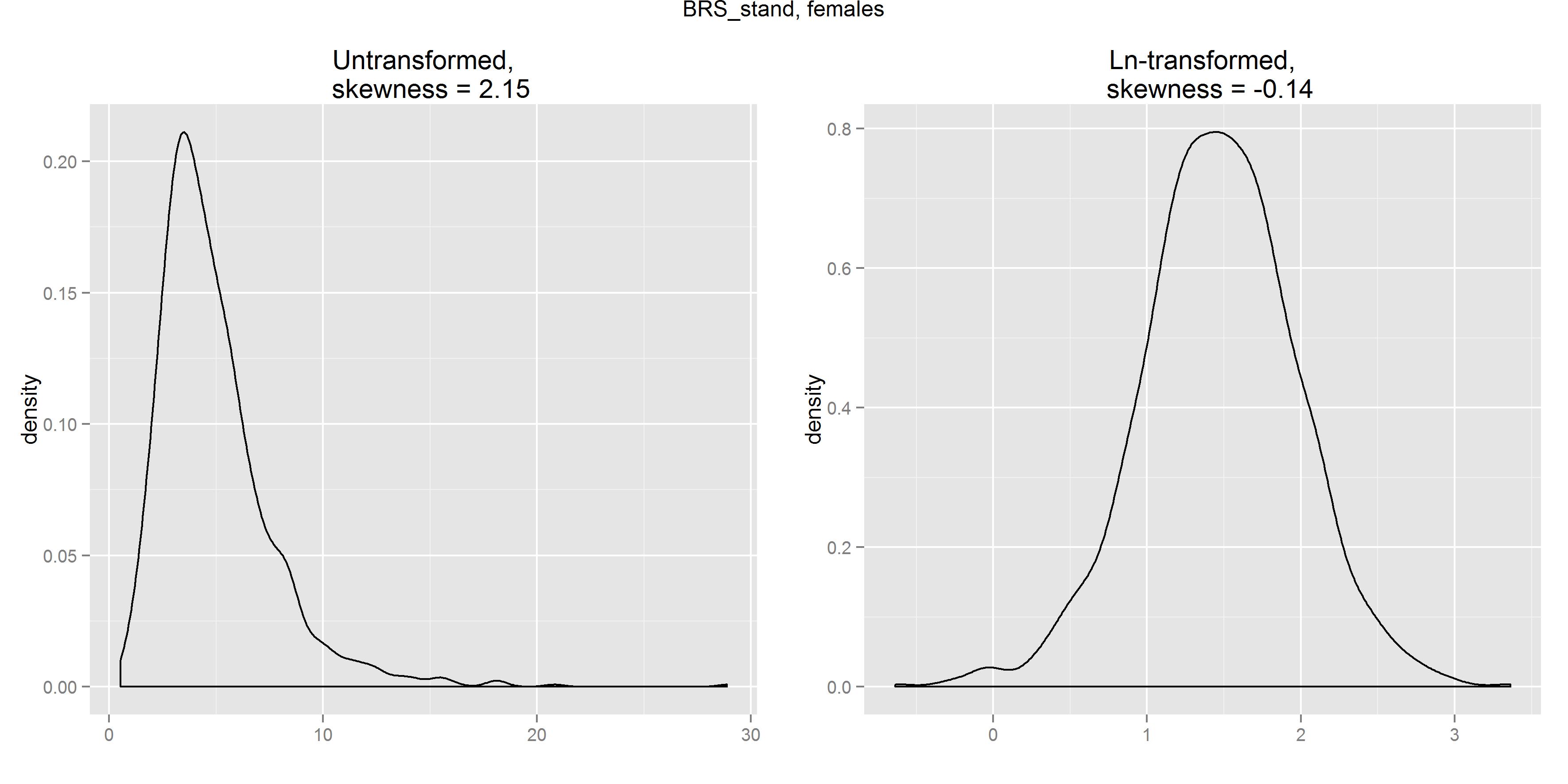

Supplement: S1 File — (ZIP) [file pone.0161604.s001.zip › BRS_stand_females_transformation_effect.jpg]

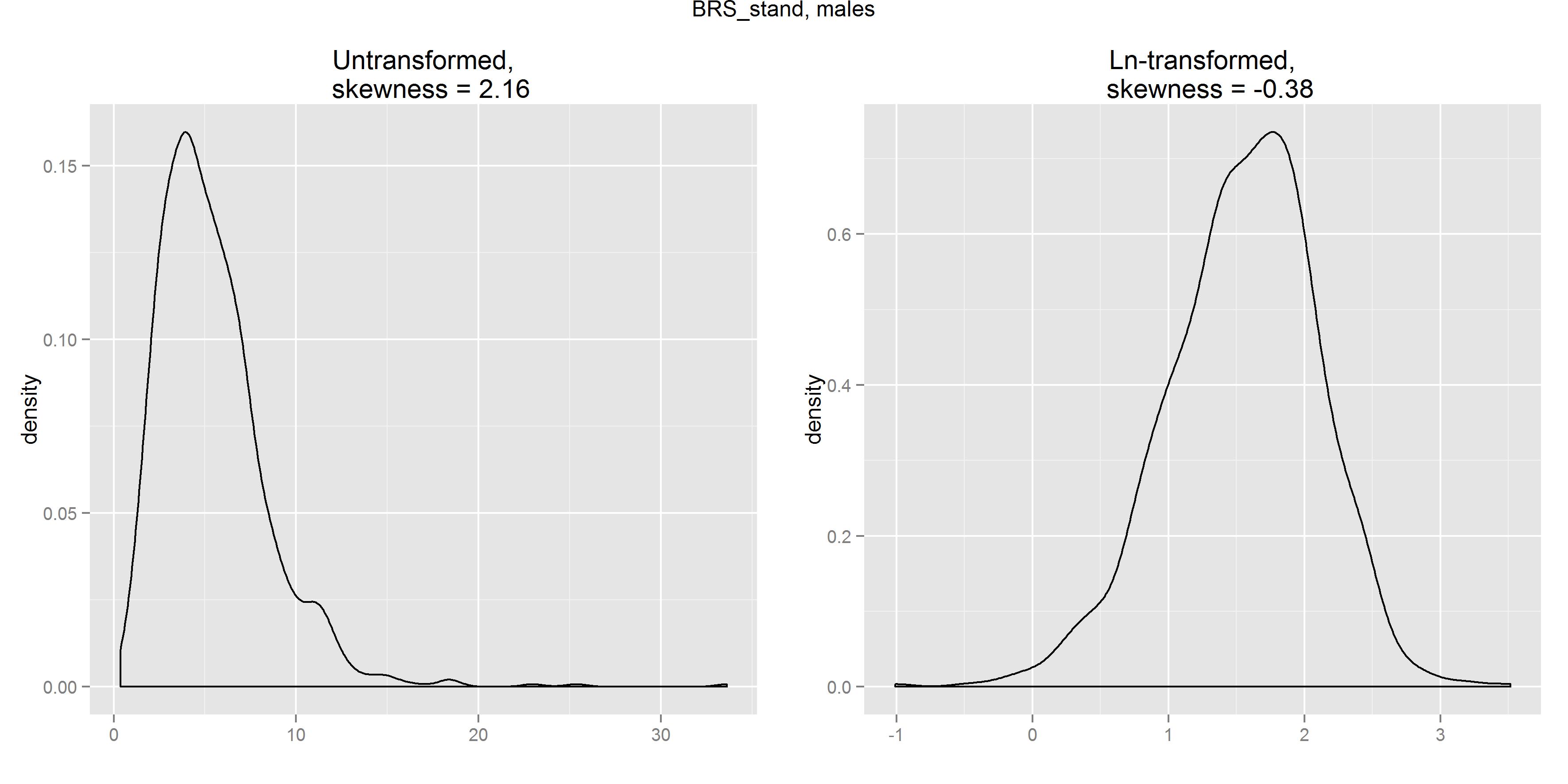

Supplement: S1 File — (ZIP) [file pone.0161604.s001.zip › BRS_stand_males_transformation_effect.jpg]

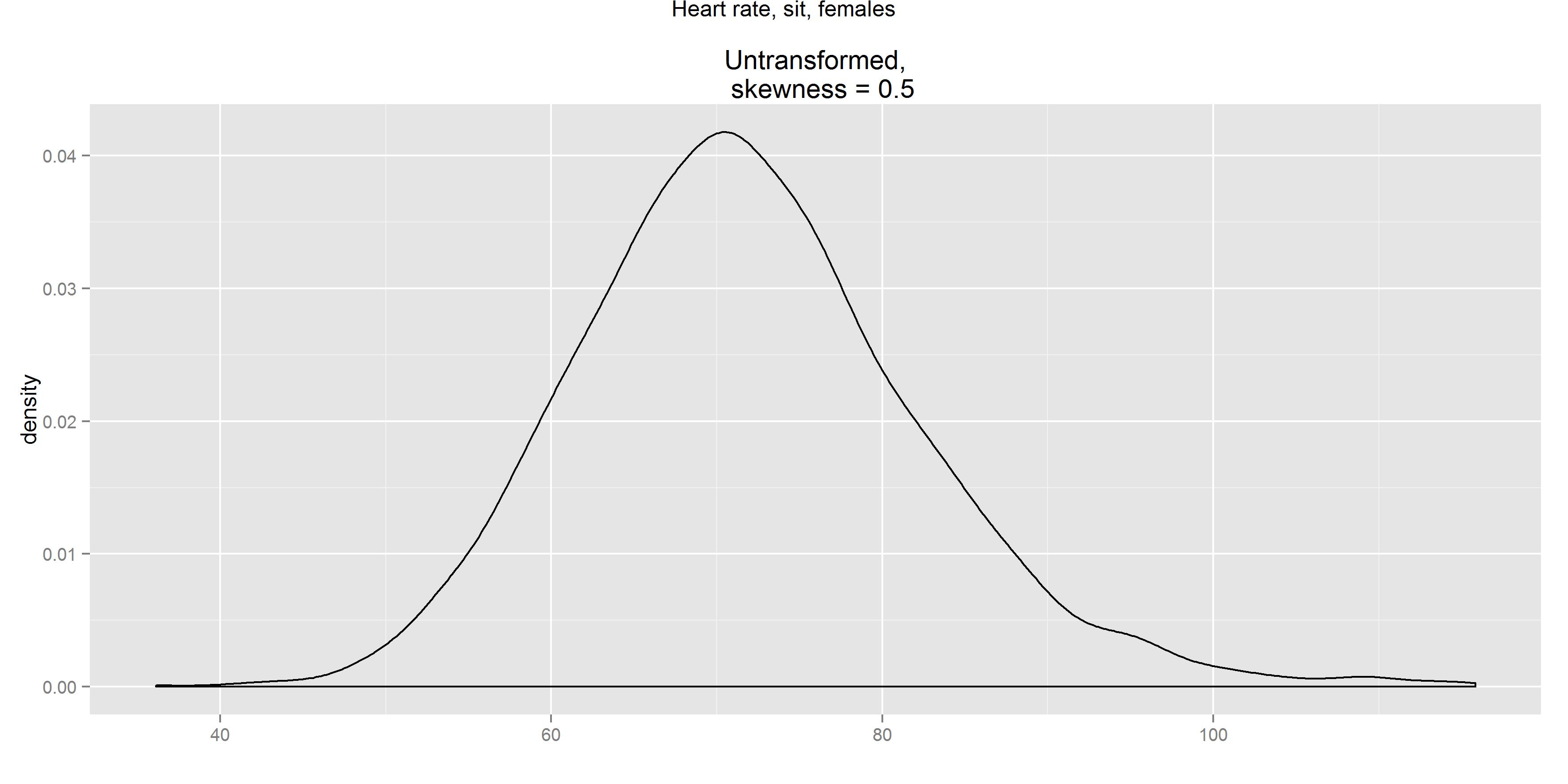

Supplement: S1 File — (ZIP) [file pone.0161604.s001.zip › Heart rate, sit_females_transformation_effect.jpg]

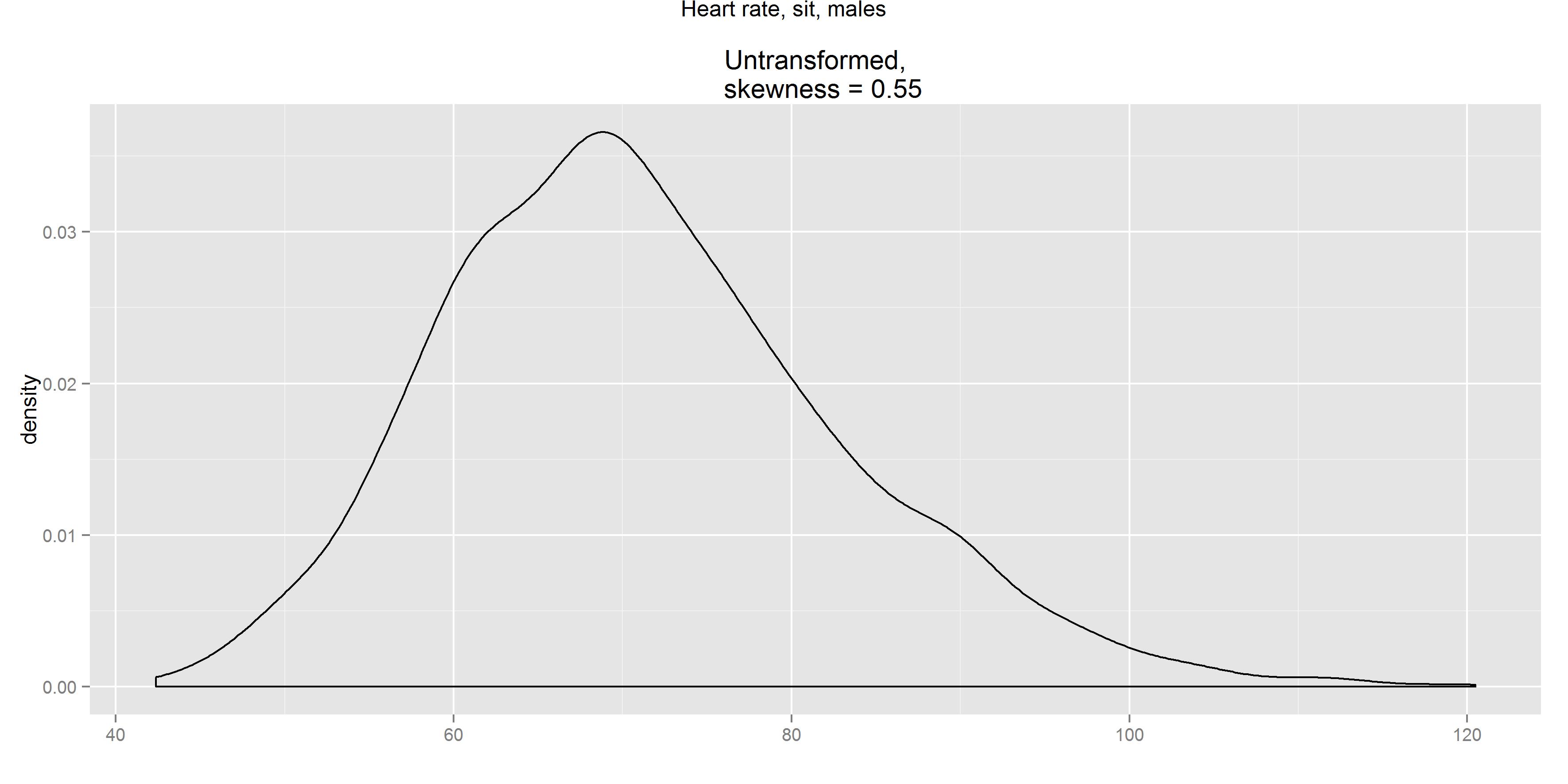

Supplement: S1 File — (ZIP) [file pone.0161604.s001.zip › Heart rate, sit_males_transformation_effect.jpg]

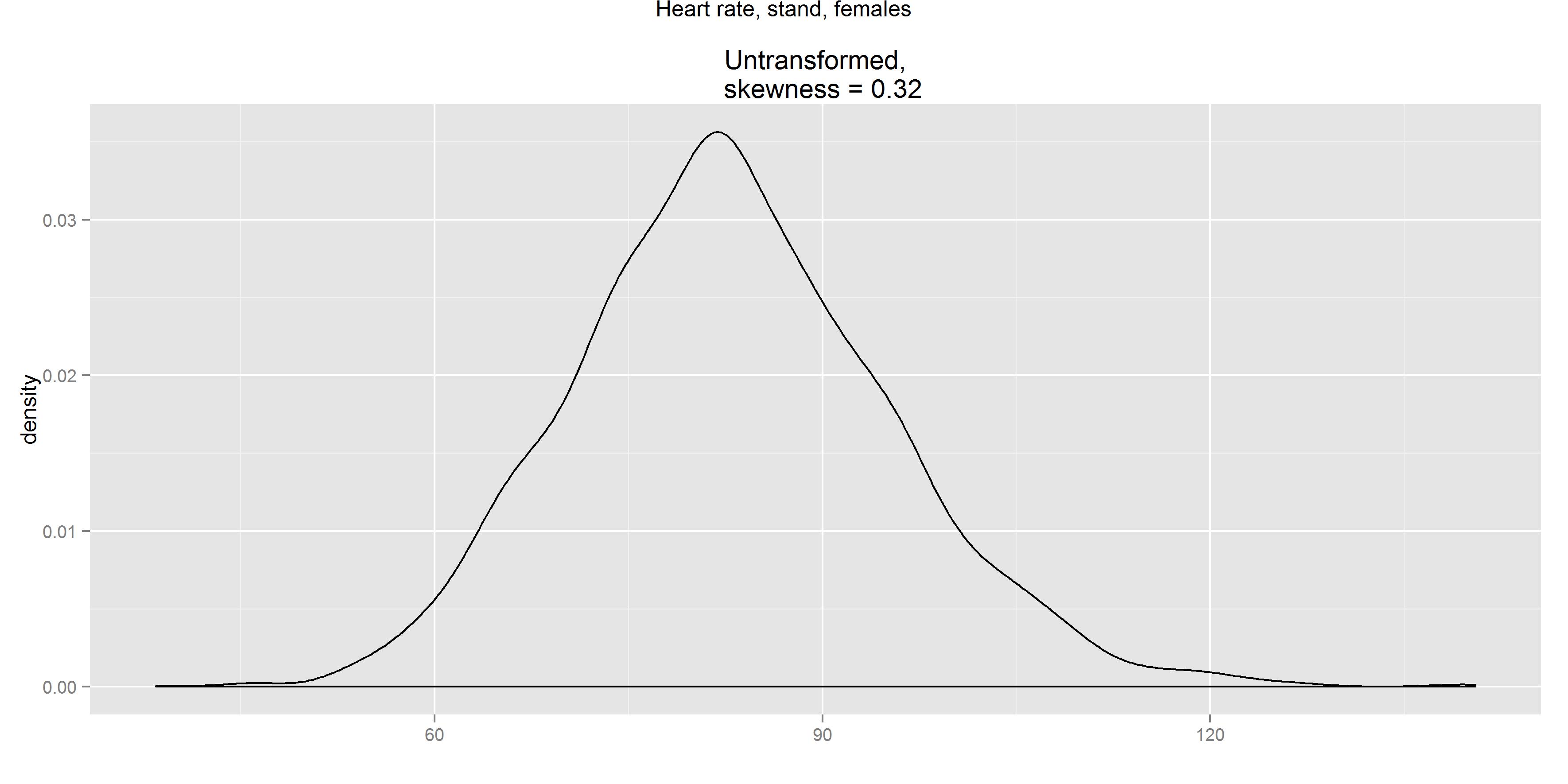

Supplement: S1 File — (ZIP) [file pone.0161604.s001.zip › Heart rate, stand_females_transformation_effect.jpg]

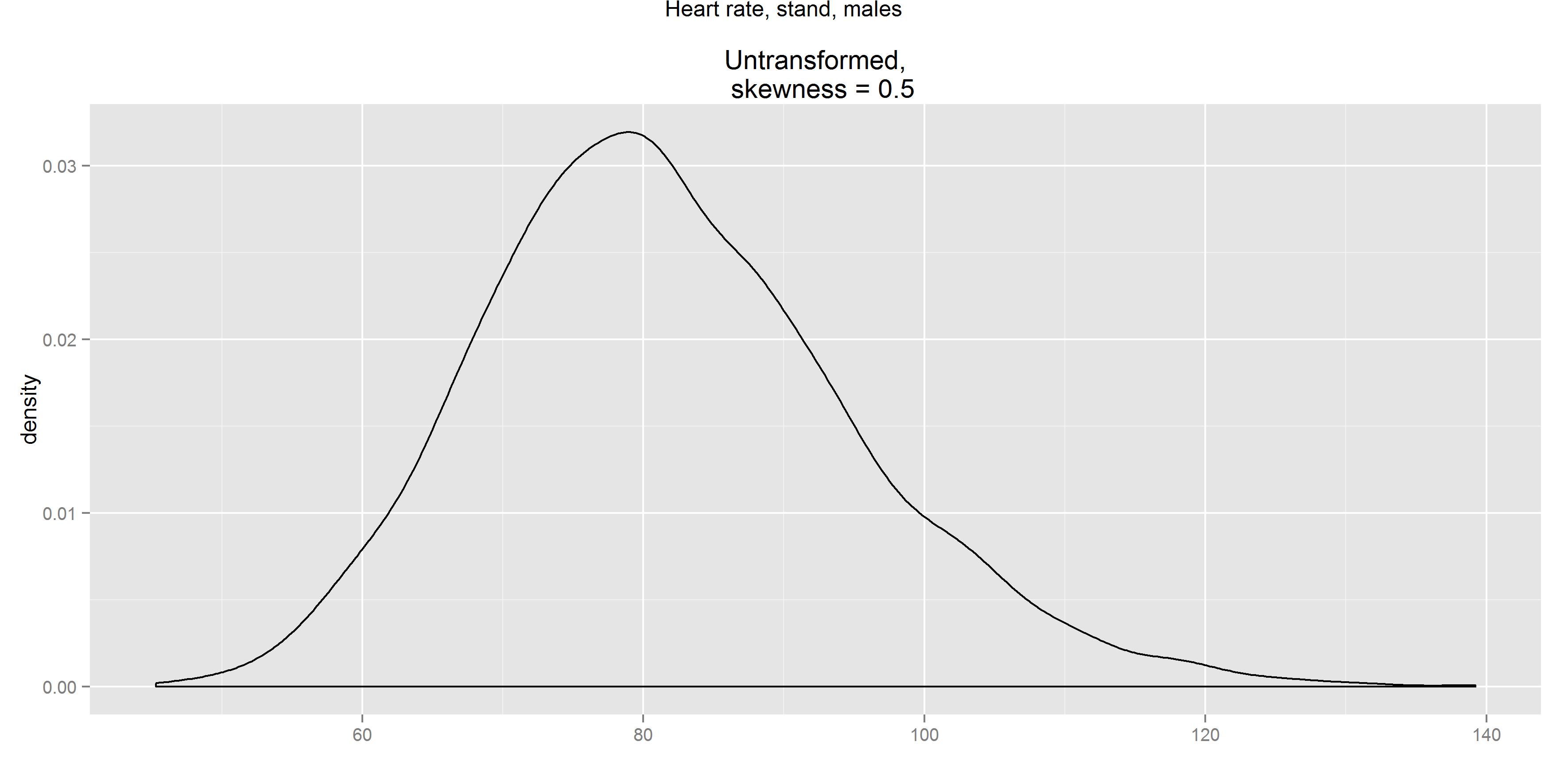

Supplement: S1 File — (ZIP) [file pone.0161604.s001.zip › Heart rate, stand_males_transformation_effect.jpg]

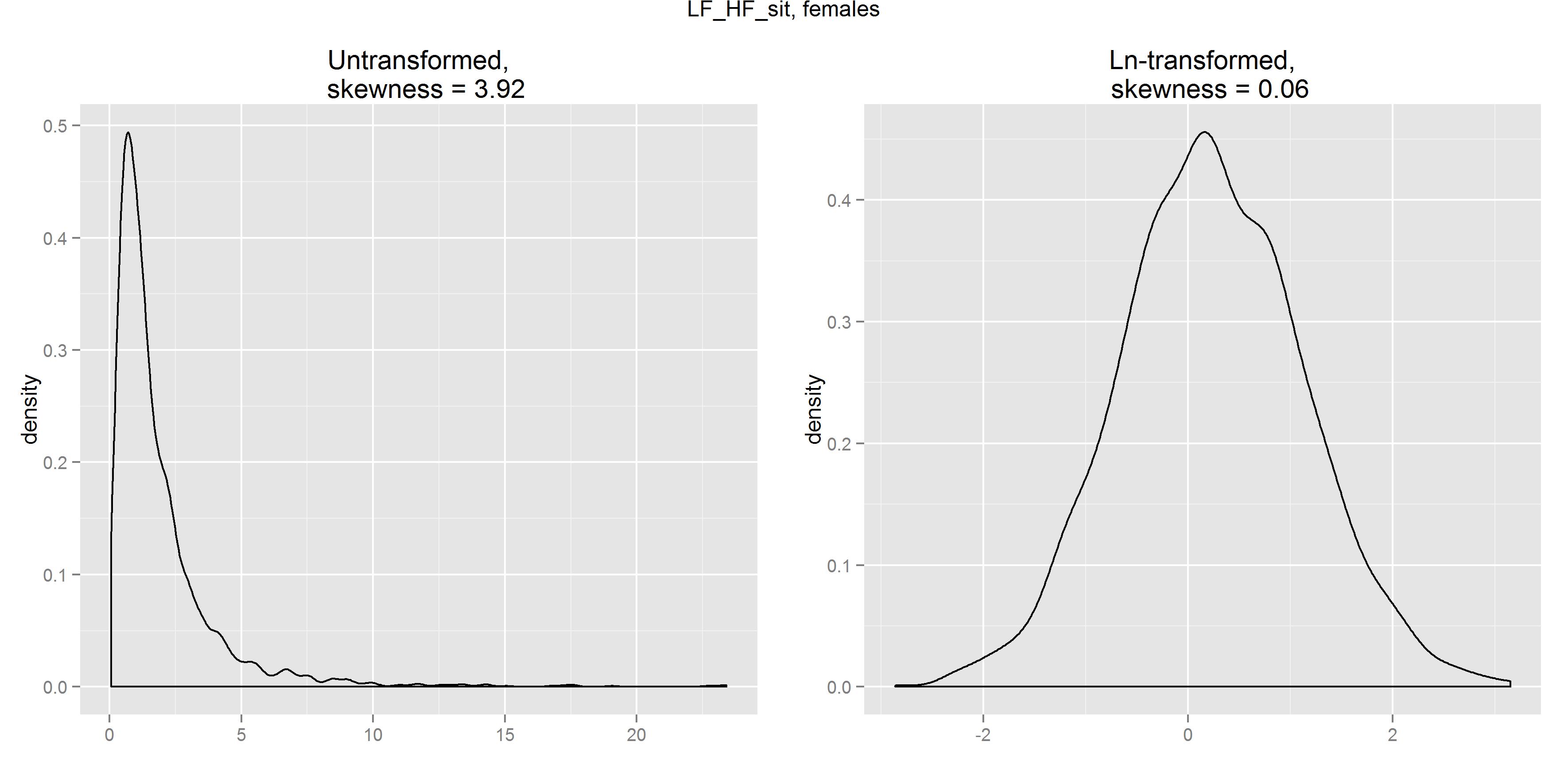

Supplement: S1 File — (ZIP) [file pone.0161604.s001.zip › LF_HF_sit_females_transformation_effect.jpg]

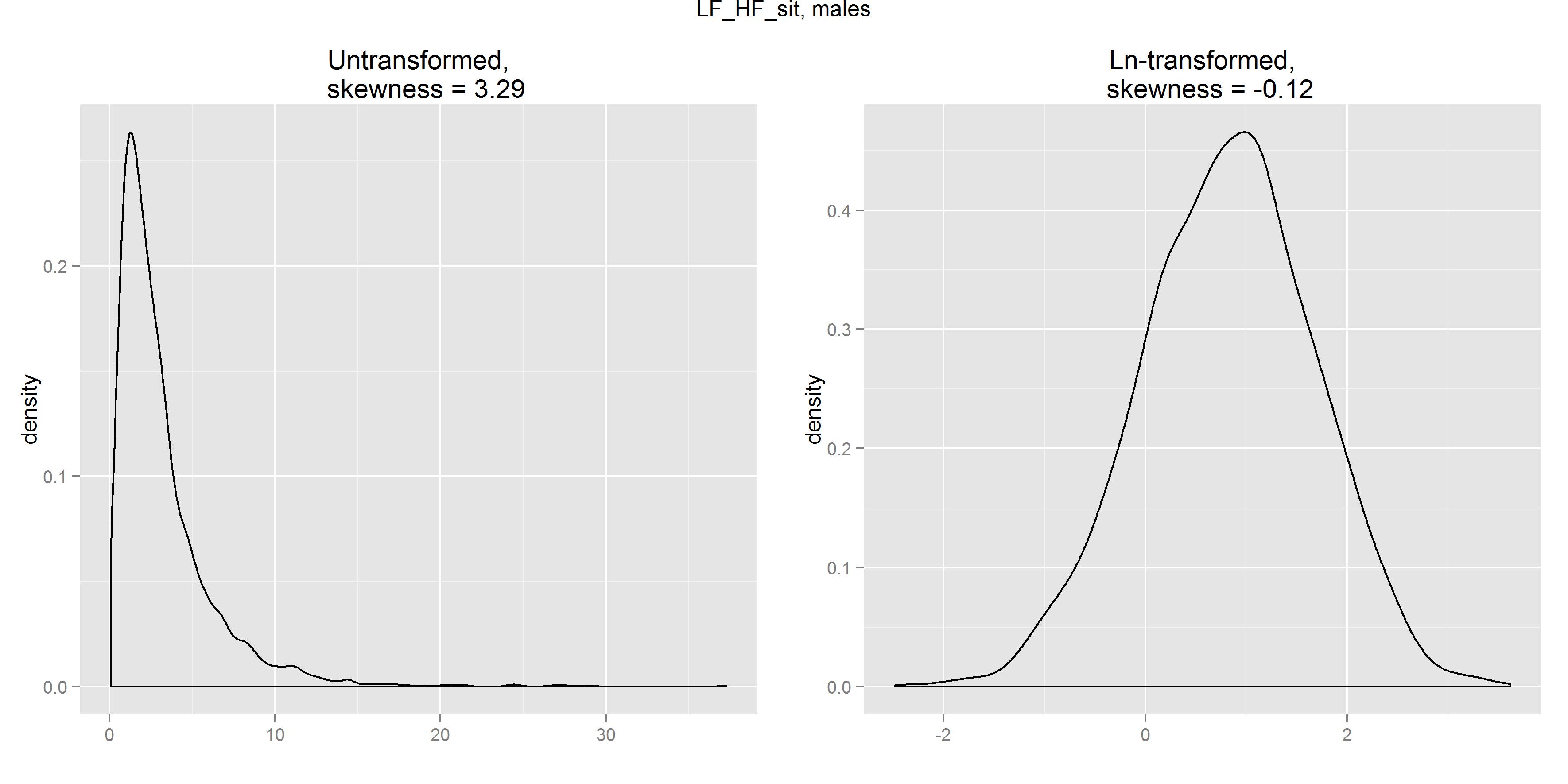

Supplement: S1 File — (ZIP) [file pone.0161604.s001.zip › LF_HF_sit_males_transformation_effect.jpg]

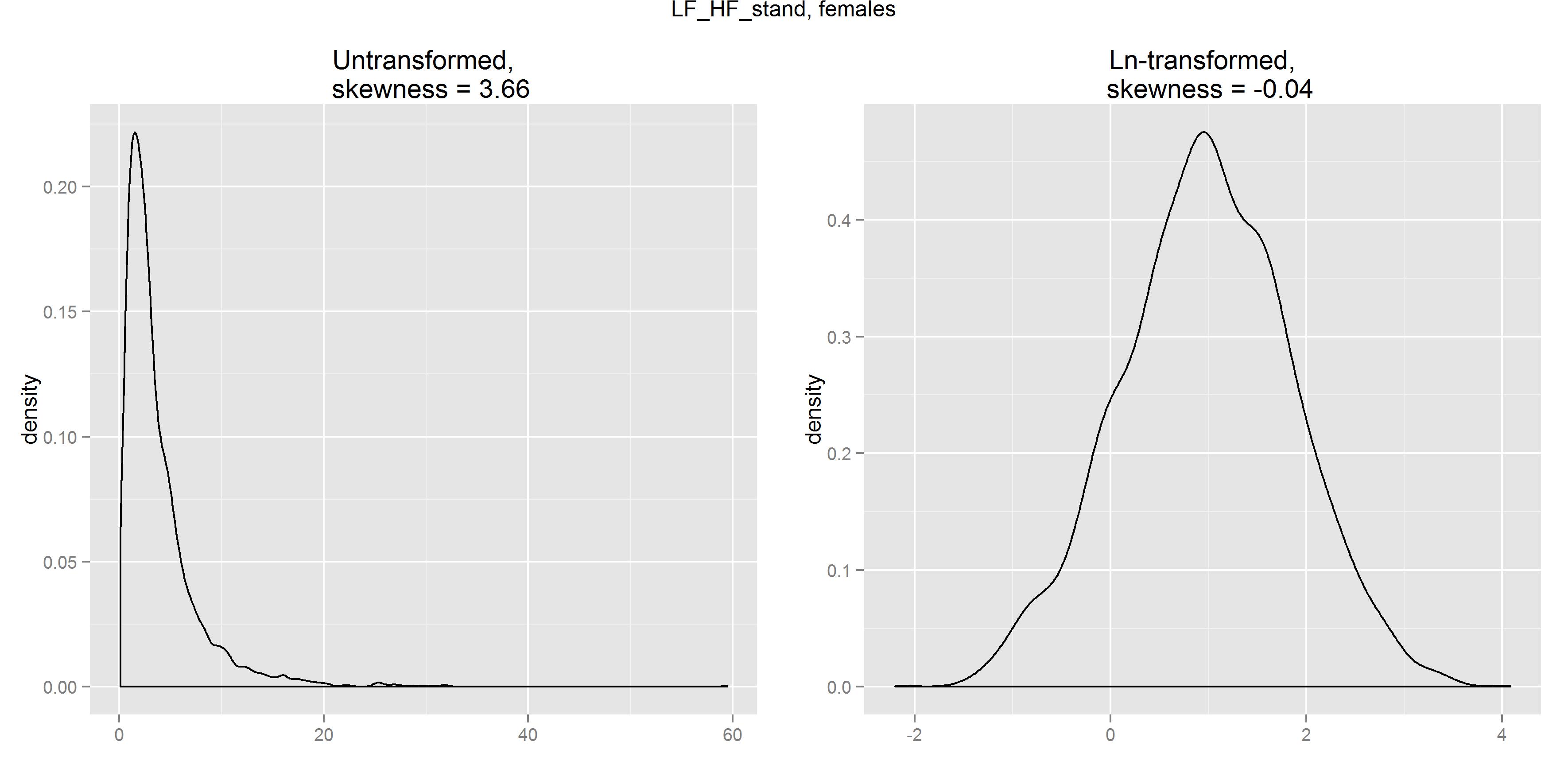

Supplement: S1 File — (ZIP) [file pone.0161604.s001.zip › LF_HF_stand_females_transformation_effect.jpg]

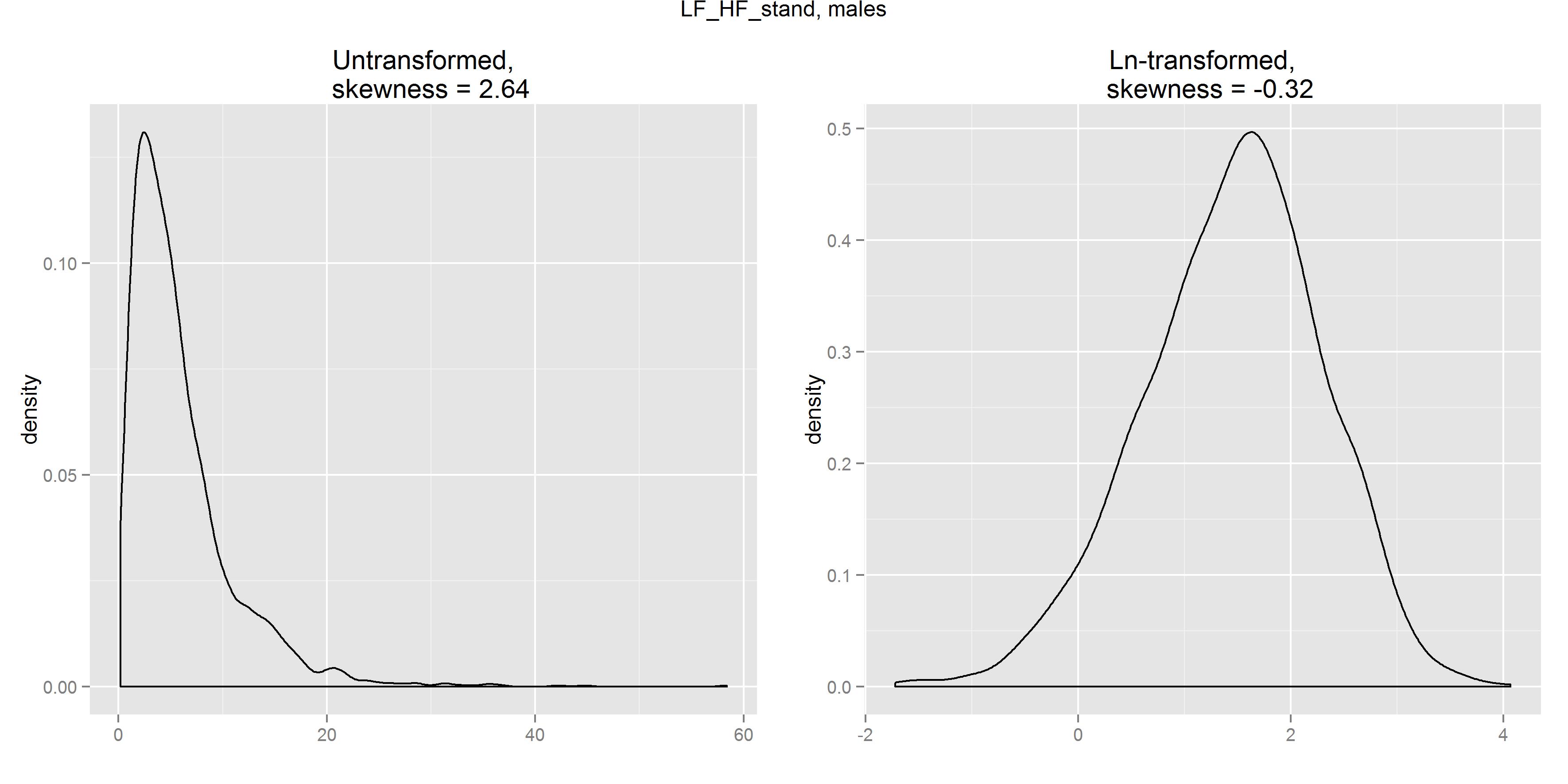

Supplement: S1 File — (ZIP) [file pone.0161604.s001.zip › LF_HF_stand_males_transformation_effect.jpg]

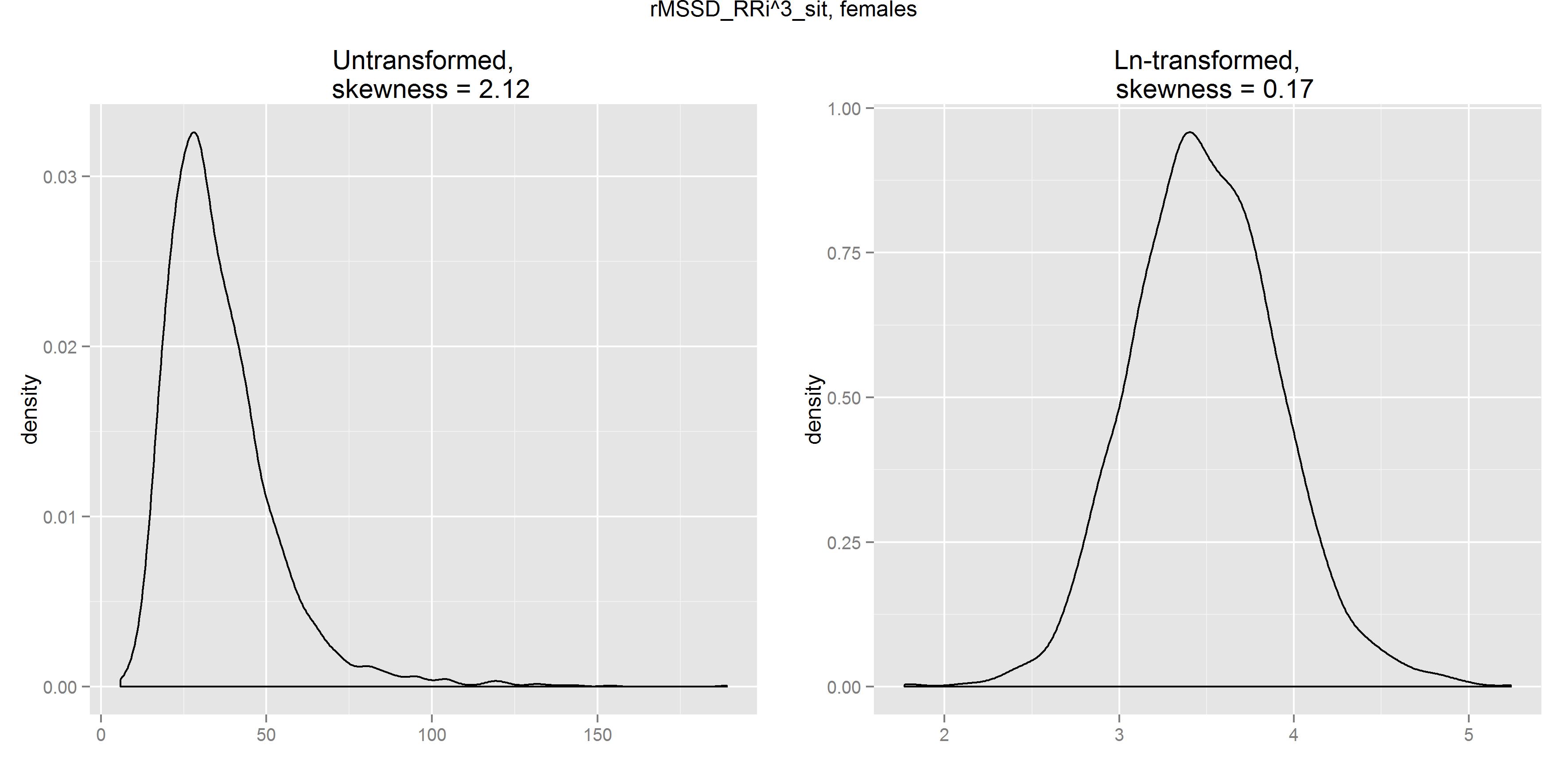

Supplement: S1 File — (ZIP) [file pone.0161604.s001.zip › rMSSD_RRi^3_sit_females_transformation_effect.jpg]

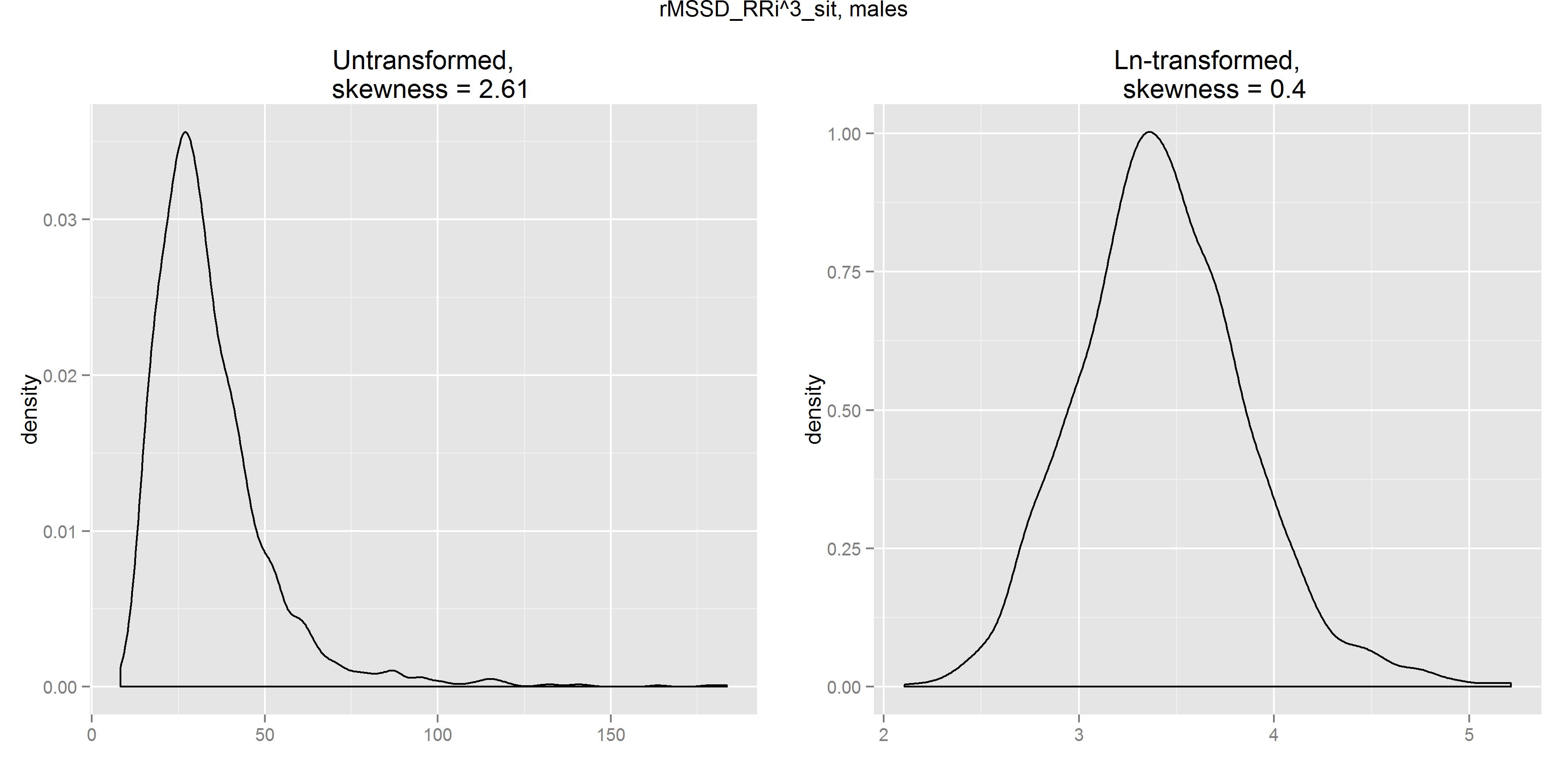

Supplement: S1 File — (ZIP) [file pone.0161604.s001.zip › rMSSD_RRi^3_sit_males_transformation_effect.jpg]

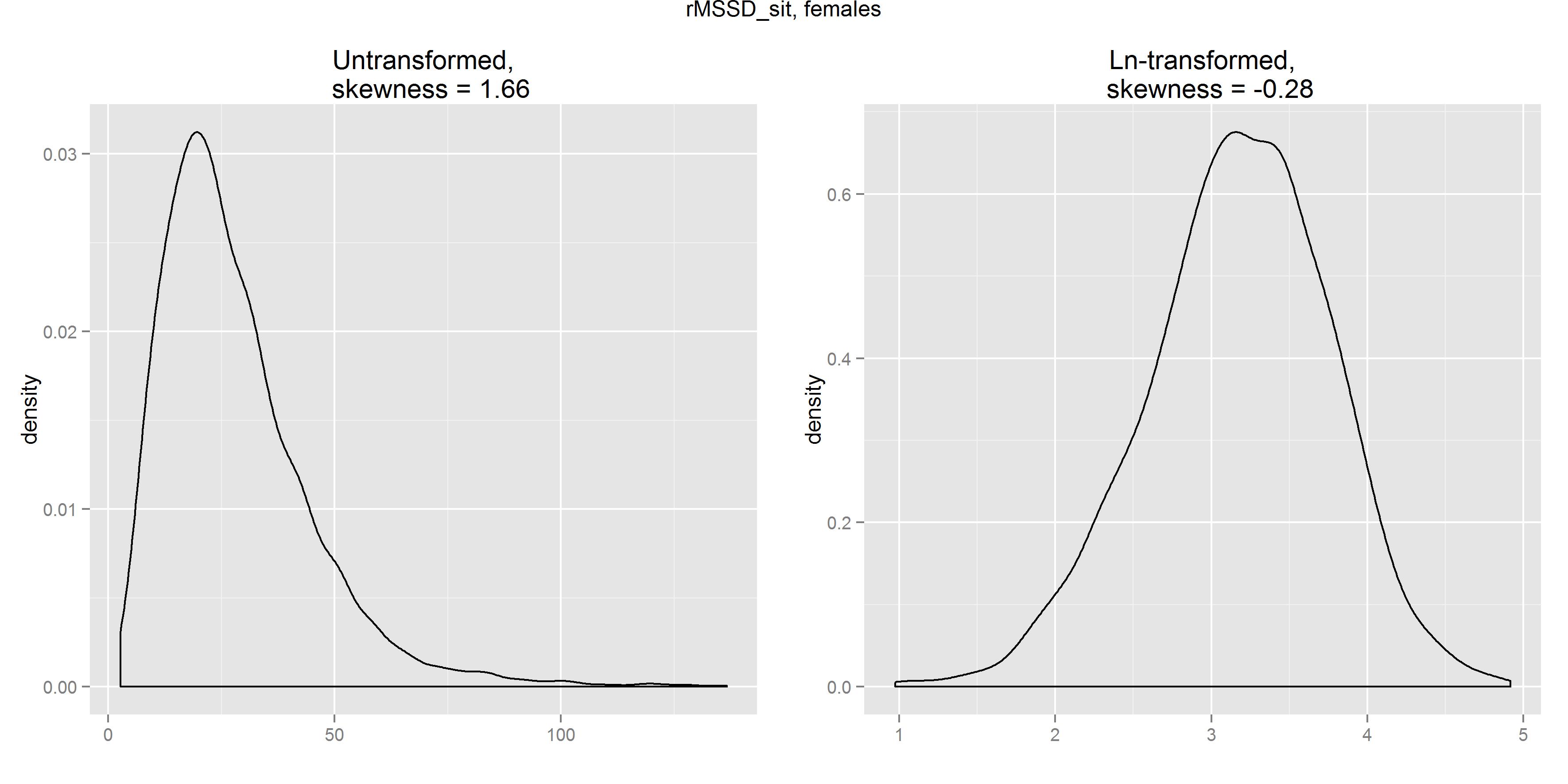

Supplement: S1 File — (ZIP) [file pone.0161604.s001.zip › rMSSD_sit_females_transformation_effect.jpg]

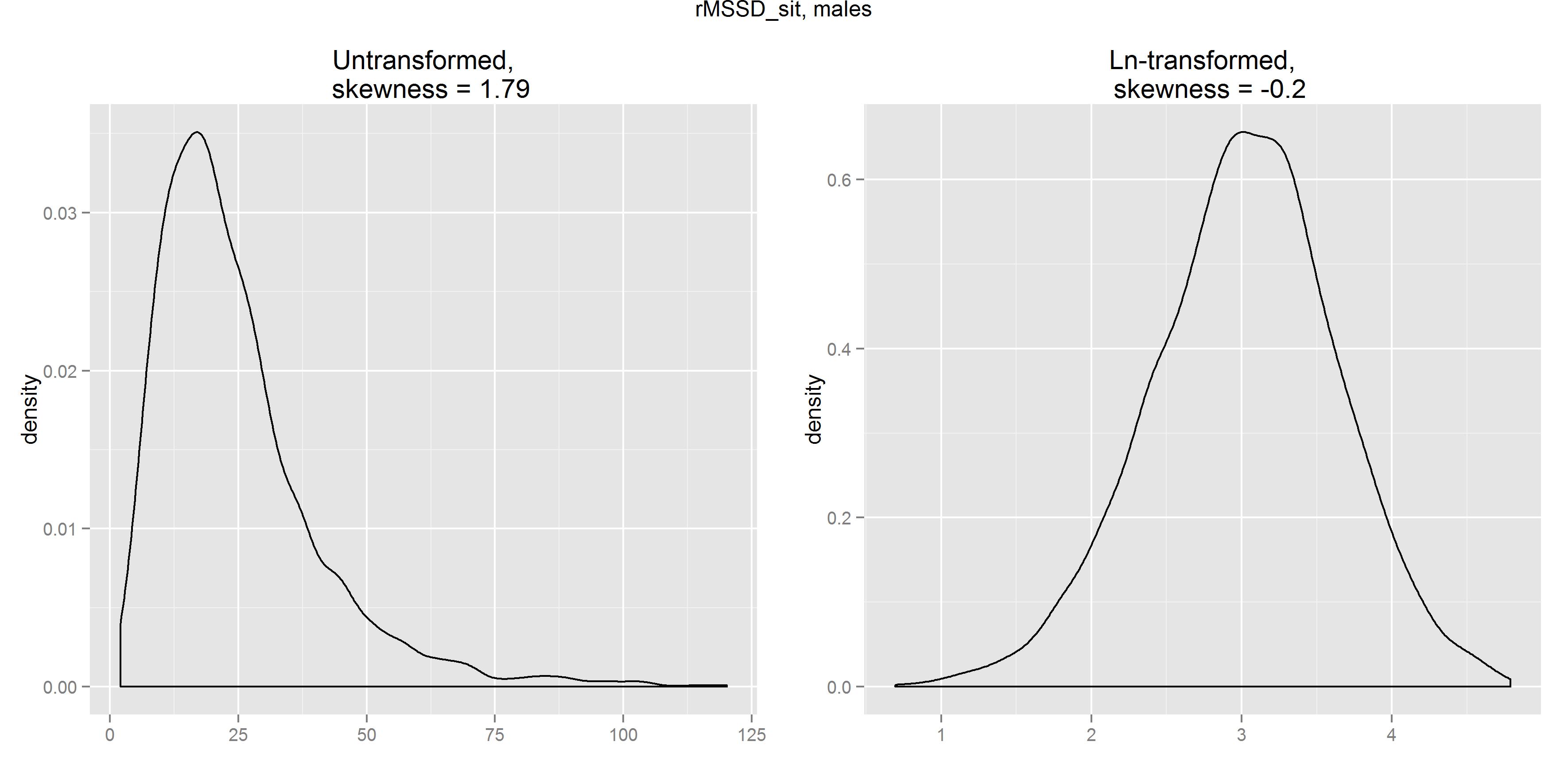

Supplement: S1 File — (ZIP) [file pone.0161604.s001.zip › rMSSD_sit_males_transformation_effect.jpg]

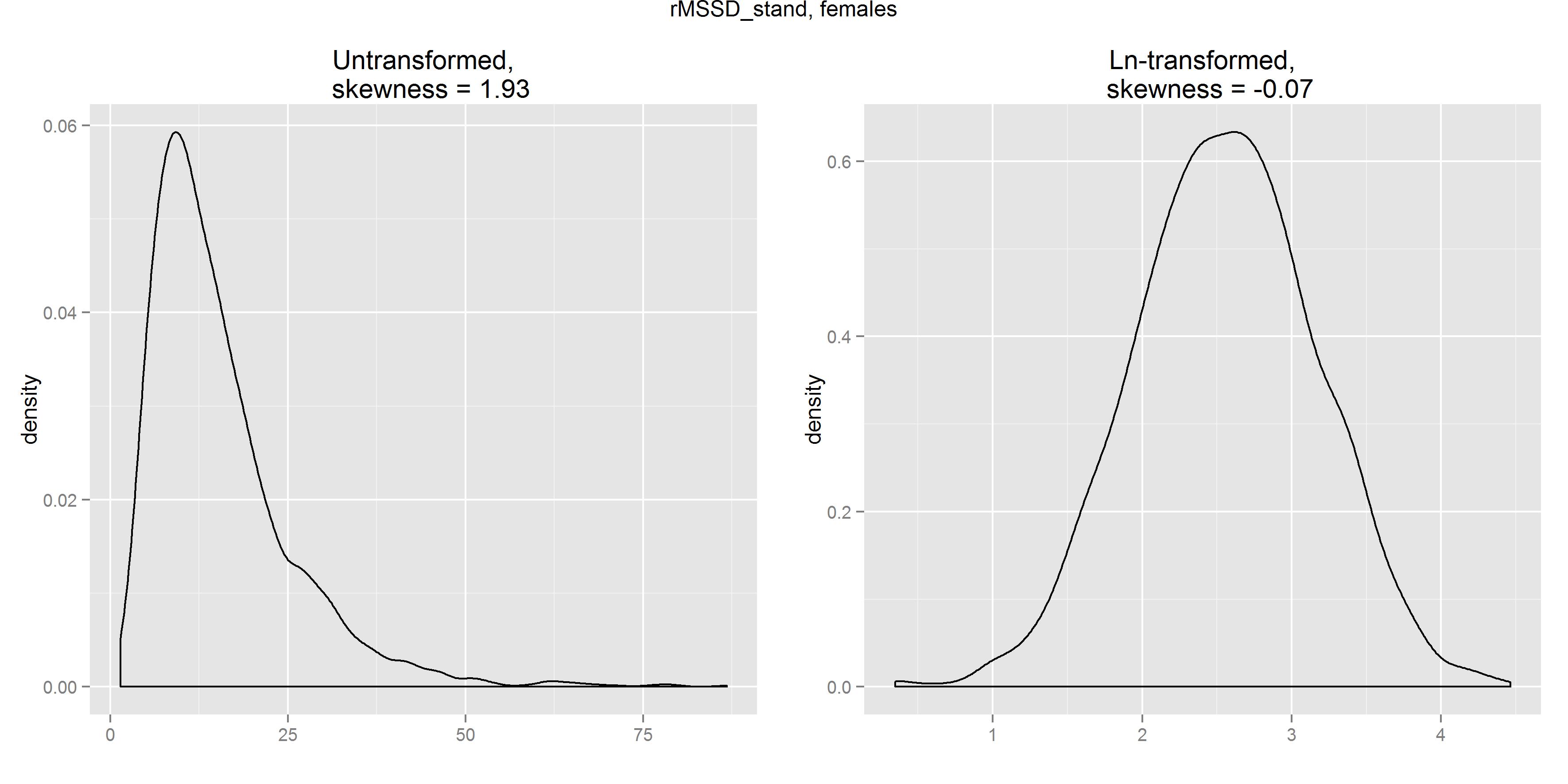

Supplement: S1 File — (ZIP) [file pone.0161604.s001.zip › rMSSD_stand_females_transformation_effect.jpg]

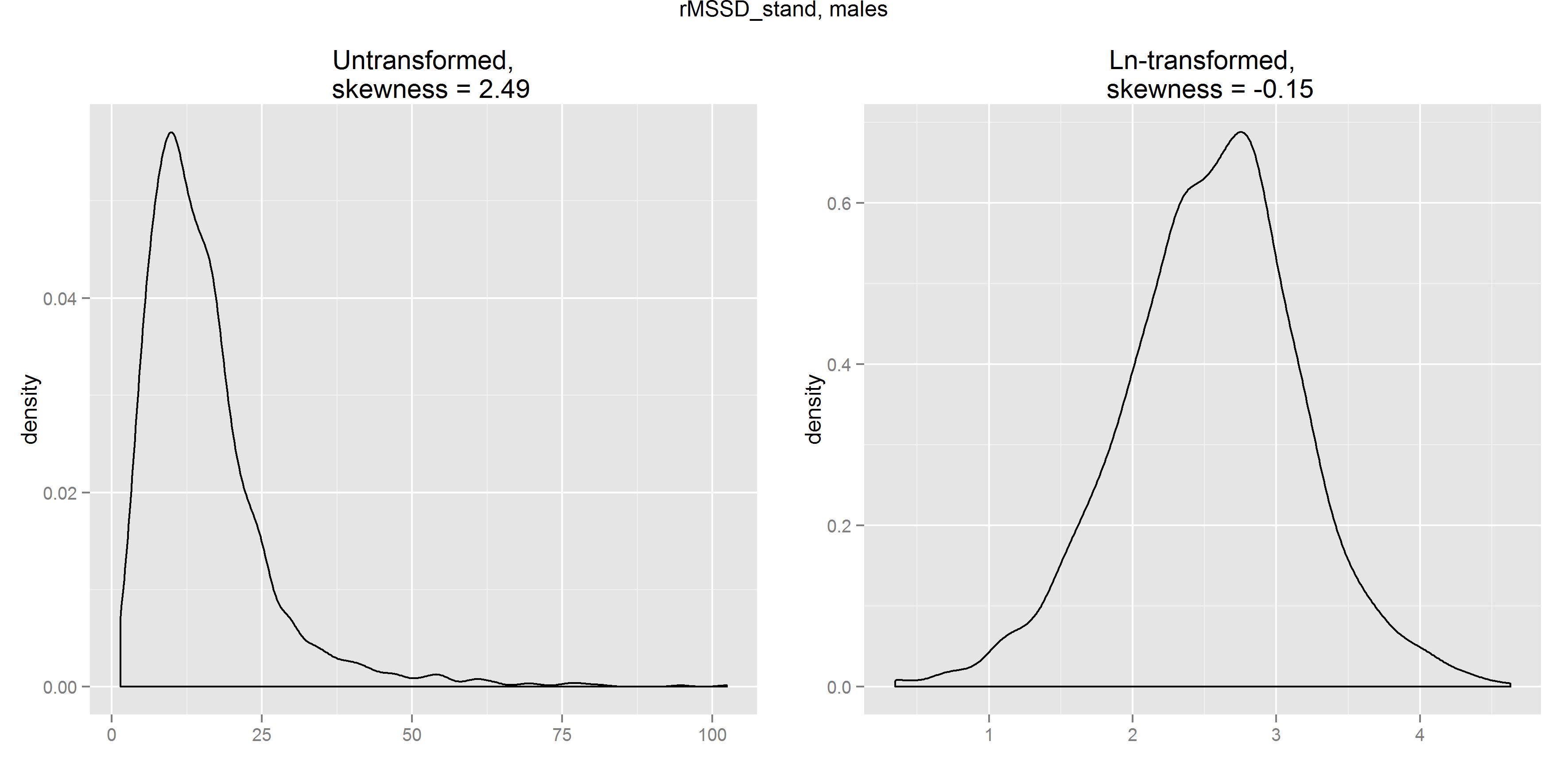

Supplement: S1 File — (ZIP) [file pone.0161604.s001.zip › rMSSD_stand_males_transformation_effect.jpg]
