# Supplementary material for: Association between Birth Characteristics and Cardiovascular Autonomic Function at Mid-Life
Source: PLoS One. 2016 Aug 23;11(8):e0161604. doi: 10.1371/journal.pone.0161604 (PMC4994955; doi:10.1371/journal.pone.0161604)
Supplement: S1 Table — (PDF) [file pone.0161604.s002.pdf]

SKEWNESS AND KURTOSIS VALUES OF CARDIOAUTONOMIC VARIABLES

Table 1. Skewness and kurtosis values of cardioautonomic variables in men.

	**Before ln-transformation**		**After ln-transformation**	
	**Skewness**	**Kurtosis**	**Skewness**	**Kurtosis**
Sitting				
HR	0.553	0.401	N/A	N/A
rMSSD	1.788	4.697	-0.202	0.156
rMSSD/RRi^3	1.904	5.632	0.292	0.417
LF/HF	3.293	19.072	-0.122	0.053
BRS	1.994	7.561	-0.230	0.632
Standing				
HR	0.502	0.426	N/A	N/A
rMSSD	2.492	10.228	-0.154	0.474
rMSSD/RRi^3	2.614	12.134	0.400	0.611
LF/HF	2.644	12.909	-0.321	0.172
BRS	2.166	11.446	-0.376	0.701

Table 2. Skewness and kurtosis values of cardioautonomic variables in women.

	**Before ln-transformation**		**After ln-transformation**	
	**Skewness**	**Kurtosis**	**Skewness**	**Kurtosis**
Sitting				
HR	0.497	0.849	N/A	N/A
rMSSD	1.659	4.661	-0.281	0.194
rMSSD/RRi^3	4.685	74.813	0.100	1.160
LF/HF	3.922	23.950	-0.063	0.060
BRS	1.489	3.233	-0.093	0.133
Standing				
HR	0.319	0.520	N/A	N/A
rMSSD	1.928	6.009	-0.068	0.041
rMSSD/RRi^3	2.118	8.576	0.172	0.521
LF/HF	3.663	26.934	-0.040	0.094
BRS	2.156	9.526	-0.143	0.696
