# Supplementary material for: Association between Birth Characteristics and Cardiovascular Autonomic Function at Mid-Life
Source: PLoS One. 2016 Aug 23;11(8):e0161604. doi: 10.1371/journal.pone.0161604 (PMC4994955; doi:10.1371/journal.pone.0161604)
Supplement: S2 Table — (DOCX) [file pone.0161604.s003.docx]

**MULTICOLLINEARITY DIAGNOSTICS FOR STATISTICAL MODELS**

**Table 1.** Multicollinearity diagnostics for statistical model with seated lnRMSSD and birth weight in men.

|  | Collinearity statistics | |
| --- | --- | --- |
|  | Tolerance | VIF |
| Birth weight | 0.813 | 1.230 |
| Father’s socioeconomic status 1 | 0.488 | 2.050 |
| Father’s socioeconomic status 2 | 0.463 | 2.159 |
| Father’s socioeconomic status 3 | 0.537 | 1.861 |
| Z-score maternal age | 0.410 | 2.442 |
| Z-score maternal age squared | 0.658 | 1.520 |
| Z-score maternal height | 0.001 | 1258.192 |
| Z-score maternal height squared | 0.614 | 1.627 |
| Z-score maternal weight | 0.000 | 6129.888 |
| Z-score maternal weight squared | 0.009 | 106.008 |
| Z-score maternal BMI | 0.000 | 5114.069 |
| Z-score maternal BMI squared | 0.014 | 70.783 |
| Maternal smoking | 0.924 | 1.083 |
| Parity 1 | 0.455 | 2.197 |
| Parity 2 | 0.658 | 1.519 |
| Adult height | 0.019 | 52.147 |
| Adult weight | 0.004 | 257.794 |
| Adult BMI | 0.005 | 212.821 |
| Adult waist-hip ratio | 0.507 | 1.972 |
| Adult mean systolic blood pressure | 0.293 | 3.415 |
| Adult mean diastolic blood pressure | 0.274 | 3.655 |
| Adult glycated hemoglobin | 0.863 | 1.159 |
| Adult fasting glucose | 0.801 | 1.249 |
| Adult fasting total cholesterol | 0.067 | 14.999 |
| Adult fasting high-density cholesterol | 0.295 | 3.392 |
| Adult fasting low-density cholesterol | 0.083 | 12.086 |
| Adult fasting triglycerides | 0.347 | 2.882 |
| Adult alcohol consumption | 0.908 | 1.102 |
| Adult smoking status | 0.871 | 1.148 |
| Adult sufficiency of sleep | 0.962 | 1.040 |
| Adult sitting time | 0.963 | 1.038 |
| Adult physical activity 1 | 0.644 | 1.554 |
| Adult physical activity 2 | 0.690 | 1.449 |

**Table 2.** Multicollinearity diagnostics for final statistical model with seated lnRMSSD and birth weight in men without low-density cholesterol, maternal weight, and adult weight in men.

|  | Collinearity statistics | |
| --- | --- | --- |
|  | Tolerance | VIF |
| Birth weight | 0.822 | 1.216 |
| Father’s socioeconomic status 1 | 0.488 | 2.047 |
| Father’s socioeconomic status 2 | 0.464 | 2.154 |
| Father’s socioeconomic status 3 | 0.538 | 1.859 |
| Z-score maternal age | 0.411 | 2.434 |
| Z-score maternal age squared | 0.658 | 1.519 |
| Z-score maternal height | 0.704 | 1.419 |
| Z-score maternal height squared | 0.968 | 1.033 |
| Z-score maternal BMI | 0.568 | 1.759 |
| Z-score maternal BMI squared | 0.691 | 1.447 |
| Maternal smoking | 0.926 | 1.080 |
| Parity 1 | 0.455 | 2.195 |
| Parity 2 | 0.659 | 1.517 |
| Adult height | 0.692 | 1.446 |
| Adult BMI | 0.483 | 2.070 |
| Adult waist-hip ratio | 0.511 | 1.956 |
| Adult mean systolic blood pressure | 0.293 | 3.409 |
| Adult mean diastolic blood pressure | 0.274 | 3.649 |
| Adult glycated hemoglobin | 0.872 | 1.147 |
| Adult fasting glucose | 0.802 | 1.246 |
| Adult fasting total cholesterol | 0.712 | 1.405 |
| Adult fasting high-density cholesterol | 0.630 | 1.588 |
| Adult fasting triglycerides | 0.588 | 1.700 |
| Adult alcohol consumption | 0.910 | 1.099 |
| Adult smoking status | 0.887 | 1.127 |
| Adult sufficiency of sleep | 0.963 | 1.039 |
| Adult sitting time | 0.964 | 1.037 |
| Adult physical activity 1 | 0.646 | 1.548 |
| Adult physical activity 2 | 0.692 | 1.445 |

The change of the dependent variable (measure of cardiovascular autonomic function) and the independent birth variable and the sex altered the multicollinearity diagnostics very little. In all of our final statistical models all VIF values were <5.
